# Evaluation der Umsetzung der Masern- und COVID-19‑Impfpflichten in Gesundheitsämtern und Gesundheitseinrichtungen: 2 Fallstudien

**DOI:** 10.1007/s00103-025-04027-3

**Published:** 2025-03-25

**Authors:** Nora Schmid-Küpke, Leonard Kranz, Eva Rehfuess, Ole Wichmann, Julia Neufeind

**Affiliations:** 1 Fachgebiet Impfprävention, STIKO, Abteilung für Infektionsepidemiologie, Robert Koch-Institut, Seestraße 10, 13353 Berlin, Deutschland; 2 Institut für Medizinische Informationsverarbeitung Biometrie und Epidemiologie, Medizinische Fakultät, Ludwig-Maximilians-Universität München, München, Deutschland; 3 Pettenkofer School of Public Health, München, Deutschland

**Keywords:** Maßnahmen, Impfquotensteigerung, Sanktionen, COVID-19-Pandemie, Qualitative Interviews, Measures, Increase vaccination rates, Sanctions, Covid-19 pandemic, Qualitative interviews

## Abstract

**Hintergrund:**

In Deutschland wurden als Reaktion auf Masernausbrüche und stagnierende COVID-19-Impfquoten 2 Impfpflichten eingeführt: Im März 2020 trat das Masernschutzgesetz in Kraft; im Dezember 2021 wurde eine einrichtungsbezogene COVID-19-Impfpflicht beschlossen. Ziel dieser Studie war es, die Umsetzung beider Impfpflichten und damit verbundene Herausforderungen in Gesundheitsämtern (GÄ) sowie betroffenen Einrichtungen zu untersuchen.

**Methodik:**

Es wurden 30 semistrukturierte Expert:inneninterviews mit GÄ und weiteren Einrichtungen geführt, die jeweils aus Bundesländern mit hohen und niedrigen Impfquoten rekrutiert wurden. Die Online-Interviews fanden im Oktober und November 2022 statt, sie wurden elektronisch aufgezeichnet, wörtlich transkribiert und mittels Framework-Analyse ausgewertet.

**Ergebnisse:**

Die Masernimpfpflicht wurde bedingt durch die COVID-19-Pandemie stark verzögert umgesetzt und oftmals auf die Zeit nach Ablauf der Übergangsfrist verschoben. Bei der zeitlich begrenzten COVID-19-Impfpflicht behinderten unter anderem langwierige Verwaltungsprozesse die Umsetzung der Maßnahmen und limitierten ihre Wirksamkeit. Sanktionen wurden in beiden Fällen kaum ausgesprochen. Große Heterogenität in der Umsetzung wurde deutlich, GÄ berichteten über rechtliche Unsicherheiten bei der praktischen Umsetzung und einen fehlenden Überblick darüber, wer melden müsste. Die Impfpflichten erzeugten Zielkonflikte bspw. in Bezug auf Personalengpässe und versagte Bildungschancen.

**Diskussion:**

Die Studie identifizierte Einflussfaktoren für die erfolgreiche Umsetzung einer Impfpflicht. Eine verpflichtende Nullmeldung der Einrichtungen, einheitliche Abläufe sowie juristische Unterstützungsangebote können die Umsetzung erleichtern. Offen bleibt, wie die unter Impfpflichten auftretenden Zielkonflikte aufzulösen sind.

**Zusatzmaterial online:**

Zusätzliche Informationen sind in der Online-Version dieses Artikels (10.1007/s00103-025-04027-3) enthalten.

## Abstract (en)

**Background:**

In Germany, two vaccination mandates were introduced in response to measles outbreaks and stagnating COVID-19 vaccination rates. In March 2020, the Measles Protection Act came into effect, and in December 2021, a COVID-19 vaccination mandate for medical institutions was enacted. The aim of this study was to examine the implementation of both vaccination mandates and the associated challenges in public health authorities (PHAs) and other affected institutions.

**Methods:**

Thirty semi-structured expert interviews were conducted with PHAs and other institutions from federal states with both high and low vaccination rates. The online interviews took place in October and November 2022, were electronically recorded, transcribed verbatim, and analyzed using framework analysis.

**Results:**

The implementation of the measles vaccination mandate was delayed due to the COVID-19 pandemic and postponed several times until after the end of the transition period. For the temporary COVID-19 vaccination mandate, lengthy administrative processes were a major aspect that hindered the implementation of measures and limited its effectiveness. Sanctions were rarely imposed in either case. Implementation was characterized by significant heterogeneity; PHAs reported legal uncertainties regarding practical implementation and a lack of clarity on who should report. The mandates also created conflicting aims between the mandate and concerns over potential personnel shortages or denied educational opportunities.

**Discussion:**

This study identified key factors for the successful implementation of a vaccination mandate. Mandatory reporting, even in cases where no problems exist, standardized procedures, and legal support could facilitate implementation. However, it remains uncertain how to resolve conflicts of aims arising from vaccination mandates.

## Einleitung

Seit März 2020 gilt in Deutschland das Masernschutzgesetz (MSG, im Folgenden auch Masernimpfpflicht genannt). Das Gesetz wurde eingeführt, nachdem es in den Vorjahren nicht gelungen war, die Masernausbrüche und -fälle zu reduzieren und dadurch zur global angestrebten Masernelimination^1^ beizutragen. Das MSG sieht vor, dass Kinder in Kindertageseinrichtungen und Schulen sowie das Personal in verschiedenen medizinischen und nichtmedizinischen Einrichtungen einen Nachweis über ihre Masernimmunität erbringen [2]. Dieser Nachweis muss vor Aufnahme der Betreuung bzw. Tätigkeit vorgelegt werden. Für bereits betreute bzw. tätige Personen wurde eine Übergangsfrist festgelegt. Die Umsetzung des MSG erfolgt durch die Länder und den Öffentlichen Gesundheitsdienst (ÖGD); bei fehlendem Nachweis können Sanktionen verhängt werden (Infobox 1).

Gut anderthalb Jahre nach Inkrafttreten des MSG wurde am 10.12.2021 zusätzlich die einrichtungsbezogene COVID-19-Impfpflicht für den Gesundheitsbereich beschlossen [3]. Beschäftigte in Gesundheitseinrichtungen wie Kliniken, Pflegeeinrichtungen (PE) und Arztpraxen (AP) mussten ihren Arbeitgeber:innen einen Nachweis über eine abgeschlossene Impfserie oder einen Genesenennachweis vorlegen. Personen ohne Nachweis durften in den Einrichtungen nicht tätig sein bzw. werden. Auch bei dieser Impfpflicht waren die Länder und der ÖGD mit der Umsetzung beauftragt, bei Nichtnachweis konnten Sanktionen verhängt werden. Die COVID-19-Impfpflicht war bis zum 01.01.2023 befristet (Infobox 1).

**Abb. 1:**
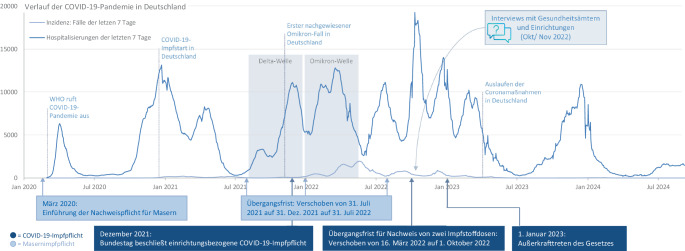
Historie der Masern- und COVID-19-Impfpflichten und Einbettung in die COVID-19-Pandemie. *Quelle*: eigene Abbildung. *Datenquelle*: Robert Koch-Institut [7]

Grundsätzlich hängt die Wirksamkeit jeder Impfpflicht entscheidend davon ab, wie und in welchem Kontext sie implementiert wird [4]. Für die Evaluation der beiden Impfpflichten wurden im Rahmen von 2 größeren Forschungsprojekten im Sinne einer *Complexity Perspective* (Intervention wird nicht isoliert betrachtet, sondern als Teil eines komplexen Systems mit entsprechenden Dynamiken) nicht nur die Auswirkung einer Impfpflicht auf das Impfverhalten untersucht [5, 6], sondern auch ihr Kontext (z. B. dominierende Virusvarianten, Abb. 1), das Setting (z. B. Ort der Intervention) und die Implementierung [5]. Ziel der vorliegenden Studie war es, die Umsetzung der beiden Impfpflichten sowie die damit verbundenen Herausforderungen in Gesundheitsämtern (GÄ) und weiteren betroffenen Einrichtungen zu untersuchen. Zudem bot sich die Gelegenheit, diese Impfpflichten vergleichend zu betrachten.

Daraus ergaben sich die folgenden Forschungsfragen:

1. Wie wurden die Impfpflichten durch die GÄ bzw. Einrichtungen umgesetzt? (Beschreibung der Prozesse)
2. Welche Herausforderungen gab es bei der Umsetzung in GÄ bzw. Einrichtungen?
3. Welchen Einfluss hatten die COVID-19-Pandemie und die COVID-19-Impfpflicht auf die Umsetzung der Masernimpfpflicht?

## Methoden

Von September bis November 2022 wurden im Rahmen einer qualitativen Studie semistrukturierte Expert:inneninterviews mit GÄ und Einrichtungen durchgeführt.

### Teilnehmende und Rekrutierung

Es wurde eine zielgerichtete Stichprobe (*Purposive Sampling, *Zusammensetzung der Stichprobe auf Grundlage von bestimmten Kriterien) der GÄ und Einrichtungen erhoben. Dabei wurde darauf geachtet, dass jeweils mindestens ein Bundesland mit hoher und eines mit niedriger Masern- bzw. COVID-19-Impfquote vertreten waren. Für die COVID-19-Impfpflicht wurden neben GÄ weitere betroffene Einrichtungen befragt, dabei wurden Einrichtungstypen mit dem größten Anteil Beschäftigter des medizinischen Sektors ausgewählt (AP, PE, Krankenhäuser (KH)) und pro Einrichtungstyp rekrutiert. Pro Einrichtungstyp sowie für die GÄ waren für die COVID-19-Impfpflicht 5 Interviews geplant, also insgesamt 20 Interviews. Für die Masernimpfpflicht waren 10 Interviews mit GÄ vorgesehen.

Die Kontaktdaten der GÄ konnten den Webseiten der Bundesländer entnommen werden. Zusätzlich wurde über die ÖGD-Feedbackgruppe des Robert Koch-Instituts (RKI) rekrutiert [8]. Weitere betroffene Einrichtungen wurden aus den für jedes Bundesland frei zugänglichen Verzeichnissen zusammengestellt; aus dieser Zusammenstellung wurde eine zufällige Stichprobe von 50 Einrichtungen gezogen. Die Einrichtungen wurden telefonisch kontaktiert und nach der für die Impfpflicht zuständigen Person gefragt. Den jeweiligen Ansprechpartner:innen wurde dann die Studie erklärt; sie wurden außerdem nach ihrer Teilnahmebereitschaft gefragt. Die Rekrutierung von AP war besonders herausfordernd, daher fand hier der Kontakt per E‑Mail statt. Die Datenschutzbeauftragte des RKI prüfte und genehmigte die Studie. Insgesamt wurden 30 Interviews mit 33 Teilnehmenden geführt (Tab. 1). Teilnehmende zur Masernimpfpflicht kamen aus 5 Bundesländern (Nordrhein-Westfalen, Baden-Württemberg, Brandenburg, Sachsen, Schleswig-Holstein), Teilnehmende zur COVID-19-Impfpflicht aus 6 Bundesländern (Schleswig-Holstein, Rheinland-Pfalz, Sachsen, Bayern, Thüringen, Brandenburg). Alle Teilnehmenden waren an der Umsetzung der jeweiligen Impfpflicht beteiligt, unterschieden sich jedoch hinsichtlich ihrer Funktion (u. a. Mitarbeitende des ärztlichen Dienstes, der Verwaltung, der Personalabteilung). Den Teilnehmenden wurde eine Incentivierung (50 € als Überweisung oder Spende) angeboten.

**Tab. 1:** Überblick über Teilnehmende (*n* = 33)

Impfpflicht	Einrichtungstyp/Gesundheitsamt			Anzahl Interviews
COVID-19	Arztpraxis (*n* = 5)	Profession	Psychotherapeut/in	2
			Arzt/Ärztin	3
		Impfquote	Hoch	2
			Niedrig	3
	Krankenhaus (*n* = 5)	Profession	Personalleitung	4
			Hygienebeauftragte/r	1
			Organisationsentwicklung	2
		Impfquote	Hoch	4
			Niedrig	3
	Pflegeeinrichtung (*n* = 5)	Profession	Einrichtungsleitung	3
			Geschäftsführung	1
			Qualitätsbeauftragte/r	1
		Impfquote	Hoch	3
			Niedrig	2
	Gesundheitsamt (*n* = 5)	Profession	Verwaltung	3
			Amtsarzt/-ärztin	2
		Impfquote	Hoch	1
			Niedrig	4
Masern	Gesundheitsamt (*n* = 11)	Profession	Verwaltung	3
			Amtsarzt/-ärztin	8
		Impfquote	Hoch	4
			Niedrig	7

### Datenerhebung und Ablauf der Interviews

Das Kernforschungsteam (NSK, LK, JN^2^) entwickelte einen semistrukturierten Leitfaden (siehe Onlinematerial). Dieser stellte die Beantwortung der Kernfragen jedes Themenblocks sicher, ermöglichte jedoch auch Nachfragen, individuelle Vertiefungen von Themen und eine flexible Reihenfolge entsprechend dem Gesprächsverlauf. Ein Framework zur Evaluation von Gesetzen half dabei, den Interviewleitfaden auf die beiden für das Forschungsvorhaben relevantesten Bereiche zu fokussieren: Machbarkeit und Funktionalität der Impfpflicht [9]. Mithilfe eines logischen Modells wurde die Einführung der Impfpflicht dann in ihrem größeren Kontext betrachtet (z. B. mögliche Wechselwirkung der Implementierung mit infektionsepidemiologischen Entwicklungen) und die Themenfelder des Leitfadens daraufhin abermals geprüft. Der Leitfaden wurde mit weiteren Forschenden pilotiert und kleinere Änderungen zur Verbesserung der Verständlichkeit vorgenommen. Nach den ersten 3 Interviews wurden der Interviewfluss, die Verständlichkeit der Fragen und der Umfang der Interviews bewertet. Es waren keine Änderungen am Leitfaden notwendig.

Vom 18.10. bis zum 21.11.2022 führten 2 in der qualitativen Forschung erfahrene Mitarbeitende (IL und SM) von Kantar Public im Auftrag des RKI die Interviews online über Cisco Webex Meetings durch. IL und SM wurden im Vorfeld zu verschiedenen Aspekten der Impfpflichten geschult. Studieninformation und Einwilligungserklärung wurden den Teilnehmenden vorab per E‑Mail zugesendet. Vor Beginn der Interviews wurden die Teilnehmenden noch einmal über die Studie aufgeklärt und willigten in die Teilnahme ein. Es folgten eine kurze Vorstellungsrunde und der Hinweis, dass die Studie im Auftrag des RKI durchgeführt wird. Zu jedem Interview war mindestens eine:r der drei Forschenden des RKI (NSK, JN und LK) anwesend, sie wurden allerdings nach der Begrüßung ausgeblendet, sodass eine 1:1-Interviewsituation entstand. Zu Beginn wurden die Teilnehmenden gebeten, ihre Rolle in der Einrichtung bzw. im GA zu erklären.

Am Ende des Interviews hatten die Teilnehmenden die Möglichkeit, offene Punkte anzusprechen, anschließend wurde die Aufnahme gestoppt und das Interview beendet. Die Interviews dauerten durchschnittlich 40 min (Spanne: 16–56 min).

### Datenanalyse

Die Tonspur der Interviews wurde aufgenommen und wortwörtlich nach Kuckartz und Rädiger [10] transkribiert sowie anonymisiert.

Die Datenanalyse erfolgte mittels Framework-Analyse und in 4 Schritten, jeweils separat für die Masernimpfpflicht (JN) und die COVID-19-Impfpflicht (LK, NSK):

1. Entwicklung einer Kategorienmatrix deduktiv aus dem Interviewleitfaden,
2. Kodierung der Daten gemäß Kategorienmatrix sowie Überprüfung der Datenkodierung, Paraphrasierung der Aussagen und geringfügige Anpassungen der Kategorienmatrix (z. B. Zusammenführung ähnlicher Kategorien),
3. Zusammenfassung der Aussagen für jede Kategorie und Untersuchung der Unterschiede nach Einrichtungstyp,
4. Zuordnung der Ergebnisse zu den Forschungsfragen (Prozesse der Umsetzung, Herausforderungen bei der Umsetzung).

## Ergebnisse

### Die Masernimpfpflicht

#### Prozesse der Umsetzung

##### Vorbereitung auf Impfpflicht, Ausstattung und Aufwand.

Die Planung der Masernimpfpflicht begann bei der Mehrheit der GÄ erst, als die COVID-19-Pandemie nachließ (Sommer 2022) und die Übergangsfrist (31.07.2022) verstrichen war (Zitat P1, Tab. 2). Die Übergangsfrist wurde dabei von der Hälfte der Befragten als Auftakt für die Umsetzung des MSG gesehen. Zum Zeitpunkt der Befragung im Herbst 2022 hatten sich die GÄ mehrheitlich und zumeist selbstständig einen Plan zur Umsetzung des MSG gemacht (bspw. Abläufe und Fristen abgesprochen, Ablaufschema und Schreiben vorbereitet und auf Rechtssicherheit geprüft; Zitat P2, P3, Tab. 2). Dabei waren die technischen Gegebenheiten hinsichtlich der Erfassung und Bearbeitung von Nachweisen entscheidend dafür, was die Umsetzung konkret an Mehrarbeit bedeutete. Die Lösungen reichten von Excel-Listen bis zu eigens entwickelter Software; das Vorgehen lief damit in einigen GÄ automatisiert, in anderen händisch ab. Für die Umsetzung des MSG waren in den GÄ unterschiedlich viele Mitarbeitende (eine Person bis hin zu größeren Teams) mit unterschiedlicher Expertise (Ärzt:innen und Verwaltungsangestellte) eingebunden; bei der Hälfte der GÄ wurde zusätzlich eingestellt.

**Tab. 2:** Schlüsselzitate für *Prozesse der Umsetzung* *(P) *und *Herausforderungen bei der Umsetzung* *(H) *des Masernschutzgesetzes

Thema	Zitat ID	Interviewter ID und Zitat
Vorbereitung auf Impfpflicht, Ausstattung und Aufwand	P1	„Quasi ab dem 1. August [2022] ist das [die Masernimpfpflicht] in den Öffentlichkeitsblickpunkt wieder gerückt … weil die Zeit gekommen war, weil wir auch durch Coronaimpfpflicht schon so ein bisschen Übung in der Bearbeitung des Impfpflichtthemas hatten, haben wir dann auch gesagt, das ist der Auftakt, wir gehen da jetzt ran.“
	P2	„Ich habe einen Prozess festgelegt, den wir jetzt noch nicht in der freien Wildbahn testen konnten, … was wir im Grunde nutzen.“
	P3	„Wir haben Vorlagen, die ich entworfen haben, teilweise auch zusammen mit den Juristen im Haus und das sind Standardgeschichten, je nach dem ist es jetzt ein Schulkind, ist es ein Kindergartenkind, ist es ein Beschäftigter … Wir machen sechs Wochen Frist, falls jemand keinen Nachweis vorgelegt hat.“
Unterstützung und Austausch	P4	„Eigentlich hieß es, mach mal, es gibt da das Gesetz, falls ihr dazu kommt, macht mal!“
	P5	„Jetzt machen es die ganzen Gesundheitsämter jeder für sich, aber letztendlich ist es ja eigentlich ein Verfahren, von daher wäre es nicht schlecht gewesen, wenn man da was an die Hand gekriegt hätte.“
	P6	„Andere Ämter habe das wieder anders ausgelegt, also aus meiner Sicht war das alles ein bisschen chaotisch und man hat sich da irgendwie so durchgewurschtelt.“
Vollständigkeit der Meldungen an das Gesundheitsamt	P7	„Also man kann die Einrichtungen, die gemeldet haben, an einer Hand abzählen.“
	P8	„Es gibt Schulen, die haben von Anfang an gemeldet, und es gibt Schulen, ein relevanter Anteil an Schulen, die haben sich noch nie gemeldet, und ob die schon mal was von Masernschutz gehört haben oder nicht, weiß ich nicht.“
	P9	„Wir konzentrieren uns jetzt erst mal auf die Schulen und Kindereinrichtungen und wenn wir dort mit dem Gros durch sind, werden wir uns sicher auch noch mal aktiv auf die anderen Einrichtungen zubewegen.“
	P10	„Ich [habe] das übernommen (habe) von einer Kollegin, … [die] das Gesetz so ausgelegt [hat], das ist eine Bringschuld der Einrichtungen und wir warten jetzt mal, was die bringen …“
Bearbeitung der Meldungen im Gesundheitsamt	P11	„Wir haben jetzt ungefähr tausend Meldungen von Schülerinnen und Schülern, von Mitarbeiterinnen oder von Menschen, die in Einrichtungen leben, von Flüchtlingen, ich tippe jetzt mal, dass wir vielleicht maximal 100 Beratungsgespräche in irgendeiner Art und Weise führen werden. Das wird wahrscheinlich ein Kraftakt werden bis zum Jahresende.“
Sanktionen	P12	„Bußgeld, Zwangsgeld konnten wir noch nicht tatsächlich aussprechen, solang diese Übergangsfrist war und die lief ja jetzt ab. In den nächsten Tagen, Wochen werden nochmal freundliche Briefe an alle rausgehen und danach gibt es schon ein Flow, wie es dann weitergeht.“
	P13	„Jetzt ist es natürlich ein großer Berg, der da vor einem steht und ich glaube alle fangen jetzt an.“
	P14	„Wir hatten ein paar wenige Meldungen von Schulen oder von Einrichtungen, aber es gab ja noch immer die Übergangsfrist, d. h. außer freundlichen Briefen, außer ein paar wenigen telefonischen Beratungen gab es keine weitere Verfolgung des Masernschutzgesetzes.“
Die Pandemie verdrängt die Masernimpfpflicht	H1	„Die Masernimpfpflicht ist ja seit 2020 gesetzlich verankert, aber ich muss sagen, mit der Umsetzung und dem Auftakt zur Umsetzung, da war tatsächlich dann erst ein Gedanken daran zu fassen, als Corona überhaupt abebbte und bei uns mehr oder weniger nachgeordnet zur Coronaimpfpflicht bei uns in Angriff genommen. … Thema Masernimpfpflicht ganz ehrlich gesagt, überhaupt nicht präsent gewesen.“
	H2	„Man merkt das, nach drei Jahren ist einfach die Kraft durch und die Bereitschaft dann auch nicht mehr da, einen kleinen Schnupfen auf der Arbeit auszusitzen oder im Homeoffice, sondern sich dann doch schneller krankzumelden als das vorher war.“
Unklarheiten bei der Umsetzung	H3	„Einfach, dass man irgendetwas an die Hand bekommt, …, ich habe das auch im Austausch mit anderen Ämtern erlebt, die standen da alle so ein bisschen im Regen, jeder hat so sein eigenes Süppchen gekocht, keiner wusste so richtig wie und was mit welchem Programm.“
	H4	„Wir sind ja nicht isoliert. Wir überlegen uns jetzt ein Zwangsgeld von 1000 €. Direkt an der Kreisgrenze im anderen Land machen die vielleicht 20 € und an der südlichen Kreisgrenze machen sie vielleicht 2000 €.“
Fehlender Überblick über Einrichtungen	H5	„Da blendet man so Szenen aus und weiß, da ist so eine Dunkelkammer und hoffentlich kriege ich da auch alle.“
	H6	„Wir müssten eigentlich Listen erstellen, wenn wir das könnten und wollten, wen gibt es überhaupt? Und dann abgleichen, hat sich einer von denen gemeldet.“
Überforderung der Einrichtung	H7	„Grundsätzlich sind die Einrichtungen nicht unkooperativ gewesen, jedenfalls im Gros, die waren halt einfach total uninformiert … Ich denke mal, das wird bei denen ähnlich gewesen sein, wie bei uns, Corona hat da alles überdeckt und das ist halt einfach mehr oder weniger vergessen worden.“
	H8	„Wenn denen ein ärztliches Attest vorgelegt wird, wo ein Arzt draufschreibt, das Kind kann nicht geimpft werden, muss ich als Einrichtungsleitung schon sehr motiviert und informiert sein, um das jetzt zu hinterfragen.“
	H9	„Also schwierig finde ich an dem Gesetz, dass Laien quasi die Schulen, die Schulsekretariate oder auch die Schulleiter oder die Klassenlehrer, die müssen jetzt Atteste beurteilen.“
Arbeitsaufwand durch Impfpflicht	H10	„Es hieß, wir müssen das Masernschutzgesetz umsetzen und ich stand dann eben vor dieser Herausforderung, wie, in welcher Form, geht das überhaupt.“
Erfahrungen mit Ungeimpften – Gefälligkeitsatteste und Verzögerungstaktiken	H11	„Ein Großteil der gemeldeten Personen, das waren tatsächlich Eltern, die einfach nicht hingekriegt haben ihren Impfpass dort vorzulegen.“
	H12	„Ich denke der Anteil, den können wir noch nicht so ganz abschätzen, aber es sind auf jeden Fall Eltern dabei, die von Anfang an kundgetan haben, dass sie das aussitzen wollen und dass die Ordnungswidrigkeitsgebühr die Oma bezahlen wird.“
	H13	„Eigentlich müsste da aus meiner Sicht tatsächlich was erfolgen, denn sonst schafft man da einfach ein falsches Signal, wenn eine Familie eine Ärztin im Amt zwei Jahre beschäftigt halten kann und letztendlich geht da mit Erfolg raus, ist das schlecht.“
Konkurrierende Werte – soziale Härten	H14	„Dann sind es häufig bei uns zugewanderte Familien, da ist ein riesen Problem, die keinen Kinderarzt finden. … Das Problem ist tatsächlich, dass die ja erst in eine Kindertagesstättenbetreuung gehen können, wenn die einen ausreichenden Masernschutz nachweisen. Da kriege ich im Moment auch noch einige Anrufe von Kindertagesstätten, die sagen, gerade bei diesen Familien, die dringend auch Betreuung benötigen würden, verzögert sich das, die Plätze bleiben frei.“

##### Unterstützung und Austausch.

Die GÄ berichteten mehrheitlich von geringer bis gar keiner Unterstützung durch Land und Bund (Zitat P4, Tab. 2). Daher erfolgte die inhaltliche Ausarbeitung des Gesetzes (z. B. Priorisierung der Zielgruppen, Festlegung von Fristen, Höhe der Bußgelder) durch jedes GA individuell (Zitat P5, Tab. 2). Die Mehrheit der GÄ hat sich regelmäßig mit anderen GÄ zur Umsetzung des Gesetzes ausgetauscht, aufgrund der COVID-19-Pandemie war der Austausch jedoch teilweise stark reduziert (Zitat P6, Tab. 2).

##### Vollständigkeit der Meldungen an das GA.

Die GÄ berichteten mehrheitlich, die Meldungen durch Einrichtungen seien noch nicht flächendeckend erfolgt (Zitat P7, Tab. 2). Das Ausmaß der Meldungen variierte sowohl nach Einrichtungstyp als auch zwischen einzelnen Einrichtungen (Zitat P8, Tab. 2). So hätten bspw. manche Schulen von Anfang an gemeldet, andere noch nie. GÄ forderten mehrheitlich aktiv Nachweise ein, wobei der Fokus auf Schulen und Kitas lag (Zitat P9, Tab. 2). Andere GÄ berichteten, auf Meldungen gewartet und diese chronologisch abgearbeitet zu haben (Zitat P10, Tab. 2).

##### Bearbeitung der Meldungen in den GÄ.

Die GÄ berichteten mehrheitlich, dass sie den Meldungen der Einrichtungen über fehlende Nachweise nachgehen. Die GÄ sind dabei unterschiedlich weit, haben jedoch mit Ausnahme eines GA alle bereits Aufforderungsschreiben verschickt. Mehrheitlich berichteten die GÄ von einem Verzug bei der Bearbeitung von Meldungen (Zitat P11, Tab. 2). Für die Vorlage eines Nachweises kommunizierten die GÄ unterschiedlich strenge Fristen (bspw. 2 Wochen bis 2 Monate).

##### Sanktionen.

Mehrheitlich haben GÄ noch keine bzw. kaum Sanktionen verhängt*.* Einige GÄ berichteten, dass Sanktionen in den kommenden Monaten folgen könnten (Zeitpunkt der Interviews: Oktober/November 2022), sobald die erforderlichen Fristen abgelaufen sind (Zitat P12, P13, P14, Tab. 2). Mehrfach wurde darauf hingewiesen: Bei Neuaufnahmen in Kindertagesstätten und Neuanstellungen in Einrichtungen ist ein Nachweis zwingend, folglich dürfte es hier auch keine Sanktionen geben. Für bereits Beschäftigte oder Betreute galt die Übergangsfrist, d. h., Sanktionen waren erst nach dem 31.07.2022 zu erwarten.

#### Herausforderungen bei der Umsetzung

##### Die Pandemie verdrängt die Masernimpfpflicht.

Mehrheitlich berichteten die GÄ, dass die Umsetzung des MSG aufgrund der COVID-19-Pandemie und zusätzlich durch die COVID-19-Impfpflicht depriorisiert wurde (Zitat H1, Tab. 2). Die Planung zur Umsetzung begann in einigen GÄ erst nach dem Abklingen der Pandemie. Einige GÄ berichteten, dass während der Pandemie viele Mitarbeitende überlastet waren und nun krankheitsbedingt länger ausfielen (Zitat H2, Tab. 2). Andere GÄ berichteten, durch das Auslaufen der COVID-19-Impfpflicht rückte die Masernimpfpflicht aber wieder in den Fokus. Bei einzelnen GÄ konnten zudem Abläufe der COVID-19-Impfpflicht übernommen werden und Kolleg:innen, die mit dieser beschäftigt waren, bei der Umsetzung des MSG unterstützen.

##### Unklarheiten bei der Umsetzung.

Einige GÄ kritisierten, dass Vorlagen und Vorgaben (bspw. für Fristen) gefehlt hätten, um das MSG einheitlich umzusetzen. Die fehlende externe Unterstützung bedeutete, dass alle GÄ eigene lokale Lösungen finden mussten und ein „Flickenteppich“ der Umsetzungen entstand (Zitat H3, Tab. 2). Die so zwischen den GÄ entstandenen Unterschiede im Umgang mit dem MSG könnten den Bürger:innen schwer zu erklären sein und auch Präzedenzfälle schaffen (Zitat H4, Tab. 2). Passend dazu gab die Hälfte der Befragten an, v. a. Fragen zur juristisch sicheren Gestaltung von Abläufen zu haben.

##### Fehlender Überblick über Einrichtungen.

Die GÄ berichteten, keinen Überblick über die Anzahl der meldepflichtigen Einrichtungen gehabt zu haben; darüber hinaus gab es keine Pflicht zur Nullmeldung (Zitat H5, Tab. 2). GÄ können nur die Meldungen bearbeiten, die auch über die Einrichtungen eingehen. Sie können nicht unterscheiden, ob eine Einrichtung bisher noch nicht gemeldet hat oder ob dort alle Nachweise vorliegen und keine Mitarbeitenden an das GA gemeldet werden müssen (Zitat H6, Tab. 2). Gleichzeitig wurde deutlich, dass die Einrichtungen bisher nicht flächendeckend gemeldet hatten. Einige GÄ teilten die Vermutung, dass es noch viel mehr Menschen gibt, die keinen Nachweis haben und nie gemeldet oder kontrolliert wurden.

##### Überforderung der Einrichtung.

Einige GÄ vermuteten, dass die Einrichtungen über ihre Aufgabe im Rahmen des MSG nicht Bescheid wussten (Zitat H7, Tab. 2). Sie berichteten, dass Einrichtungen viele Fragen hatten und von ihren Pflichten überrascht waren. Zudem waren Einrichtungen, so berichteten einige GÄ, insbesondere mit der Überprüfung von Nachweisen und Attesten überfordert (z. B. serologischer Nachweis, Kontraindikationen; Zitat H8, Tab. 2). Die GÄ regten an, dass die Prüfung der Atteste nicht durch die Einrichtungen und damit zumeist durch medizinische Laien, sondern direkt durch die GÄ erfolgen sollte (Zitat H9, Tab. 2).

##### Arbeitsaufwand durch Impfpflicht.

Einige GÄ beklagten die fehlende oder unzureichende Software für die Erfassung und Bearbeitung von Meldungen. Bestehende Excel-Listen (und andere Programme) seien sehr umständlich und würden viel Verwaltungsarbeit erzeugen (H10, Tab. 2). Sie äußerten den Wunsch nach einer einfacheren Verwaltung von Meldungen, um manuelle Arbeit (bspw. Listen übertragen) zu vermeiden. Andere GÄ berichteten hingegen von Software, die z. B. automatisiert Anschreiben an Bürger:innen verschickt und so die Arbeit erleichtert.

##### Umgang mit Ungeimpften – Gefälligkeitsatteste und Verzögerungstaktiken.

Die GÄ machten mehrheitlich die Erfahrung, dass viele Personen, bei denen ein Nachweis fehlte, lediglich vergessen hatten, sich zu impfen, oder versäumt hatten, den Nachweis vorzulegen (H11, Tab. 2). Bei den bisher eher seltenen bekannten Fällen von Impfgegner:innen kämen allerdings, so berichteten einige GÄ, auch Gefälligkeitsatteste und Verzögerungstaktiken zum Einsatz. GÄ berichteten von Ärzt:innen, die serienmäßig Atteste ausstellen, und von langwierigen Diskussionen darüber, warum die ausgestellte Kontraindikation bei einer Masernimpfung nicht gültig sei. Auch würden die Einrichtungen selbst solche Gefälligkeitsatteste möglicherweise nicht erkennen. Sie berichteten von Personen, die immer neue Wege fänden, die Impfung zu verzögern, und damit rechnen würden, die Impfung so zu umgehen (H12, Tab. 2). Ein GA empfahl, dass der Staat hier zeigen müsse, dass er sich durchsetzen kann (H13, Tab. 2).

##### Konkurrierende Werte – soziale Härten.

Einzelne GÄ berichteten von der Herausforderung, die Impfpflicht in Geflüchtetenunterkünften umzusetzen. So sei ein Betretungsverbot nicht umsetzbar, da es keine alternative Unterbringungsmöglichkeit gäbe. Einzelne GÄ berichteten, Bußgelder würden nicht eingesetzt, weil die Geflüchteten keine finanziellen Mittel hätten. Ein GA befürchtete bei zugewanderten Familien soziale Härten, wenn ein Kitaplatz aufgrund eines fehlenden Impfnachweises nicht angenommen werden kann (H14, Tab. 2).

### Die einrichtungsbezogene COVID-19-Impfpflicht

#### Prozesse der Umsetzung

##### Vorbereitung auf Impfpflicht, Ausstattung und Aufwand.

Wie die Einrichtungen sich auf die Impfpflicht vorbereiteten, hing maßgeblich von der Einrichtungsgröße und dem damit verbundenen Aufwand bei der Prüfung der Nachweise ab. So mussten sich AP in den meisten Fällen nicht auf die Impfpflicht vorbereiten, PE und KH hatten mehrheitlich Konzepte für die Umsetzung vor Ort erarbeitet, z. B. hatten sie ein eigenes Impfmanagement geschaffen, eine Impfstraße aufgebaut oder im Vorfeld Gespräche mit impfkritischen Mitarbeitenden geführt (Zitat P1, P2, Tab. 3). Für die GÄ waren die technischen Voraussetzungen (Software, Portal oder Excel-Listen), die Vorgaben im GA bzw. Bundesland und die personellen Ressourcen sehr unterschiedlich und für die Vorbereitung entscheidend. Einigen GÄ wurde eine Infrastruktur bereitgestellt, andere GÄ mussten sich selbst behelfen (Zitat P3, Tab. 3). In den GÄ und weiteren Einrichtungen waren unterschiedlich viele Mitarbeitende mit unterschiedlicher Expertise in die Umsetzung der Impfpflicht eingebunden (ärztliche Leitung, Jurist:in, Verwaltung etc.).

**Tab. 3:** Schlüsselzitate für *Prozesse der Umsetzung* *(P) *und *Herausforderungen bei der Umsetzung* *(H) *der COVID-19-Impfpflicht

Thema	Zitat ID	Interviewter ID und Zitat
Vorbereitung auf Impfpflicht, Ausstattung und Aufwand	P1	„… Haben uns auch in einer großen Runde, … ausgetauscht, wie wir damit umgehen, wie wir die Erfassung steuern können. Haben dann mit den Einrichtungsleitungen angefangen und haben uns Konzepte überlegt, die wir umsetzen können.“
	P2	„Und in diesem Impfportal oder in dieser Impfdatenbank haben wir dann letztendlich sämtliche Impfungen, die bei uns im Haus durchgeführt wurden, auch registriert.“
	P3	„… wollten wir dahingehend ja auch auf ein Digitalisierungstool zurückgreifen, was uns bei der Bewältigung … dieser Durchführung des Gesetzes da auch behilflich ist, dass das in geordneteren Bahnen und auch weg von der Handakte kommen … Dahingehend waren wir nicht die Vorreiter, das gebe ich zu. Bis wir dann alles hatten, ist bis Mai die Zeit vergangen.“
Unterstützung und Austausch	P4	„Also vom Bund haben wir direkt nichts gehört, zumindest nicht, was ich wüsste. … Und vom Land <<< Name des Bundeslandes >>> gab es zu dem Zeitpunkt auch noch nicht viel, außer nur die Rahmenbedingungen.“
	P5	„Jein. Seitens des Landes an der Stelle ja, da haben wir jede Unterstützung gekriegt, die wir brauchten.“
	P6	„… es gibt ja die Handreichung vom Gesundheitsministerium, die hat noch mal ein bisschen Licht ins Dunkel gebracht …“
Sammlung und Kontrolle der Nachweise in Einrichtungen	P7	„Der personelle Aufwand bei uns in der Personalabteilung war schon sehr groß. Zwischen ein und acht Mitarbeiterinnen und Mitarbeiter sind abgestellt worden, die in den Hauptzeiten dann dort neben dem täglichen Tagesgeschäft dann beschäftigt waren, das war schon sehr aufwendig bis hin zu dem Procedere der Übermittlung der Daten der nicht Geimpften, die musste alle einzeln manuell, also händisch eingegeben werden.“
	P8	„Am Anfang ist man von einfachen Exceltabellen ausgegangen, wo dann eben Name und dann die Impfdaten zum Einpflegen dann gegangen ist.“
Bearbeitung der Meldungen im Gesundheitsamt	P9	„Nicht, dass die Ersten schon zum Tätigkeitsverbot angehört werden, während andere noch nicht mal das erste Schreiben erhalten haben …“
Sanktionen	P10	(Gespräch mit MA zu angekündigter Eigenkündigung) „Das konnte aber dann im Gespräch aufgefangen werden; eben mit dem Hinweis, du wirst von uns keine Kündigung als solches, arbeitgeberseits … bekommen.“
	P11	„Die gemeldeten Personen wurden jetzt auch schon alle vom Gesundheitsamt angehört, aber es wurde kein Berufsverbot weiter ausgesprochen. Es wurde bisher immer das Verfahren eingestellt.“
	P12	„Weil, wie gesagt, das ist einfach politischer Zündstoff und wir wirklich gesagt haben, wir starten das Verfahren, aber im Hinblick auf ein Bußgeldverfahren und Tätigkeits- und Betretungsverbot gibt es von unserer Seite her nicht.“
	P13	„Weil man da schon eher sehr zurückhaltend und ängstlich ist, weil das Ganze ja doch juristisch auf dünnem Eis steht.“
Kontext Pandemie und Faktor Zeit: Enger Zeitrahmen verhindert konsequente Umsetzung der Impfpflicht	H1	„Ja, also mal ganz kurz zusammengefasst, das Gesetz ist nicht rund. Es hat seine Ecken und Kanten, es ist nicht bis zum Schluss gedacht, schon allein im Hinblick darauf, dass das Gesetz ein dreiviertel Jahr gilt und so ein Verfahren sich bis zu einem dreiviertel Jahr locker hinziehen kann.“
	H2	„Diese Kraft zu investieren und letzten Endes mit dem Blick darauf, dass wir gar nicht so weit kommen dieses Gesetz umzusetzen …“
	H3	„Es war eine einzige Mitarbeiterin, die gesagt hat, ich nehme ein Beschäftigungsverbot in Kauf, ich lasse mich auf keinen Fall impfen, was wir sehr bedauert hätten, weil das eine jahrelang sehr gut arbeitende Mitarbeiterin … ist … Da haben wir sogar überlegt, wie könnten wir die eventuell … im Homeoffice zumindest mal bis die Impfpflicht ausgelaufen ist …, wie können wir die weiter beschäftigen.“
Unklarheiten bei der Umsetzung	H4	„…, selbst wenn es eine Landesverordnung gibt oder gab, gab es ja immer, es ist nie leichte Sprache und alle sind halt keine Juristen, also was bedeutet das letzten Endes in der Übersetzung für die konkrete Herangehensweise vor Ort in den Einrichtungen.“
	H5	„… <<< Name der Landeshauptstadt>>> sagt halt immer, macht es einfach, setzt den Bußgeldbescheid. Aber im Endeffekt bleiben wir ja auf den Kosten sitzen, wenn der Richter XY dann einfach sagt, nein, wir weisen das ab, weil, ihr habt falsch entschieden. Da interessiert es dann auch keinen im Land.“
Fehlender Überblick über Einrichtungen	H6	„Also diese Lücke hätte ausgenutzt werden können oder wurde auch garantiert ausgenutzt, dessen sind wir uns auch bewusst …“
	H7	„Genau, ich denke, je kleiner so eine Einrichtung ist, desto mehr Eigenverantwortung tragen die ja dann auch, der Leiter dieser Einrichtung. Ich denke in den großen Häusern kann man sich schlecht diesen Formalismen entziehen, aber je kleiner die Einrichtung, desto möglicher halte ich es, dass man da auch mal zwei Augen zudrückt …“
Arbeitsaufwand durch Impfpflicht	H8	„Ich kann Ihnen gerne mein Überstundenkonto zeigen.“
	H9	„Also ... jetzt bei uns im Unternehmen betrachtet fand ich diese ganze Bürokratie, die damit einhergegangen ist, katastrophal.“
Umgang mit (ungeimpften) Beschäftigten: emotionale Belastungen und Spannungen	H10	„… Die jetzige Variante ist ja einfach eine Variante, die häufig einen milderen Verlauf macht. Wir reden nicht von der ersten Variante. Da [war] impfen oder nicht impfen schon lebensentscheidend manchmal. Das ist eben nicht mehr so, insofern ist die Impfpflicht natürlich noch kritischer zu sehen …“
	H11	„… und ich sage es Ihnen auch ganz ehrlich, ich weiß nicht wie ich es kommunizieren soll, dass oder eine Entscheidung, wenn es heißt, wir verlängern über den 1.1... Mir fehlt jegliche Vorstellungskraft, wie ich das den Menschen begreiflich machen soll. Wir haben draußen Festivals, wir haben Konzerte, wir haben <<< Name des Events >>> …“
	H12	„Die Mitarbeiterschaft kann natürlich nicht nachvollziehen, warum wir eine einrichtungsbezogene Impfpflicht haben und alle Mitarbeitenden sich impfen müssen und boostern müssen und es keine allgemeine Impfpflicht ausgesprochen wird, d. h., die Patienten, die gegebenenfalls nicht geimpft sind oder nicht ausreichend geimpft, werden behandelt von unseren Mitarbeitern, die sich für diese Patienten eben boostern lassen müssen. Man würde ja dann im Umkehrschluss eigentlich verlangen, dass dann auch die Patienten vollständig geimpft sind. Ich glaube, das hat einen großen Unmut verursacht.“
	H13	„… es muss auch sehr viele Anfeindungen zwischen diesen beiden Lagern gegeben haben. Insbesondere, wenn es dann auch zu Ausbruchsituationen kam, immer dieser mehr oder weniger latent oder offen geäußerte Vorwurf …“
	H14	„… jetzt lass Dich doch endlich impfen, sonst können wir mit Dir nicht weiter planen. Ansonsten wissen wir nicht, wie wir diese Dienste abdecken …“

##### Unterstützung und Austausch.

Zwei der drei Einrichtungstypen sowie alle GÄ erlebten wenig bis keine Unterstützung durch die Gesundheitsministerien oder den Bund. Zumeist wurden die Rahmenbedingungen vorgegeben und die Grundvoraussetzungen geschaffen (z. B. Einführung eines Meldeportals), die inhaltliche Ausarbeitung musste jedoch durch die Einrichtungen bzw. GÄ selbst erfolgen (Zitat P4, Tab. 3). Demgegenüber stehen Berichte einzelner KH und der Mehrheit der PE, die die Unterstützung der Ministerien positiv wahrnahmen (Zitat P5, P6, Tab. 3). Einige KH und die Mehrheit der PE berichteten, sich regelmäßig mit anderen Einrichtungen ausgetauscht zu haben. Auch unter einigen GÄ fanden regelmäßige Gespräche statt. Offene Fragen und Unklarheiten zur Impfpflicht wurden zumeist in diesen Austauschrunden und intern thematisiert.

##### Sammlung und Kontrolle der Nachweise in Einrichtungen.

Die Sammlung der Nachweise war für die Einrichtungen unterschiedlich aufwendig, je nachdem, ob bestehende digitale Strukturen (Datenbanken, Apps etc.) genutzt werden konnten. Einige PE und KH sowie alle AP berichteten, dass die Nachweise händisch gesammelt wurden (Zitat P7, P8, Tab. 3). In den Einrichtungen waren außerdem verschiedene Personen mit unterschiedlichen Kompetenzen für die Sammlung und Prüfung der Nachweise zuständig (bspw. Mitarbeitende der Personalabteilung oder des betriebsärztlichen Dienstes).

##### Bearbeitung der Meldungen im GA.

Alle GÄ hatten zum Zeitpunkt der Interviews Anschreiben an gemeldete Personen verschickt, gingen den Meldungen der Einrichtungen also nach. Die GÄ bearbeiteten die Meldungen jedoch unterschiedlich schnell (Anschreiben wurden erstmalig frühestens im Februar/März 2022 und spätestens im August 2022 verschickt), unterschiedlich strukturiert und mit unterschiedlich strengen Fristen. Die lange Dauer bis zu Sanktionen begründeten einige GÄ damit, dass Schreiben in Tranchen verschickt, Fristen beachtet und Rückmeldungen abgewartet werden mussten (Zitat P9, Tab. 3).

##### Sanktionen.

Die Einrichtungen berichteten fast einstimmig, dass keine Sanktionen wie Kündigungen und Betretungsverbote verhängt wurden. Angedrohte Eigenkündigungen konnten z. B. durch Gespräche mit den betroffenen Mitarbeitenden abgewendet werden (Zitat P10, Tab. 3). Es war außerdem möglich, einen Versorgungsengpass anzumelden und das Verfahren einzustellen, falls die betreffenden Mitarbeitenden dringend benötigt wurden (Zitat P11, Tab. 3). Einige AP wiesen darauf hin, dass es auch vonseiten der GÄ keine Nachfragen zum Impfstatus der Mitarbeitenden gab, eine Nichtumsetzung also nicht aufgefallen wäre. Auch die GÄ gaben an, bisher keine Betretungs- oder Tätigkeitsverbote ausgesprochen zu haben; teilweise war dies mit der Hausleitung von vornherein so entschieden worden (Zitat P12, Tab. 3). Einzelne GÄ berichteten zudem, keine Bußgelder verhängt zu haben (Zitat P13, Tab. 3).

#### Herausforderungen bei der Umsetzung

##### Faktor Zeit: Enger Zeitrahmen verhindert konsequente Umsetzung der Impfpflicht.

Einige der GÄ bemängelten, dass die einrichtungsbezogene COVID-19-Impfpflicht nicht konsequent umsetzbar gewesen sei. Die verwaltungsrechtlichen Vorgaben bei den Fristen und die Abläufe bei den Meldungen verbunden mit der kurzen Laufzeit der Impfpflicht (10 Monate) hätten deren Umsetzung behindert (Zitat H1, Tab. 3). Sanktionsverfahren könnten beispielsweise mehrere Monate dauern (Zitat H2, Tab. 3). Einzelne Einrichtungen berichteten, dass sich die Befürchtungen um Folgen der Impfpflicht, wie z. B. eine Verstärkung des Fachkräftemangels, nicht realisiert hätten und führten dies darauf zurück, dass kaum Sanktionen ausgesprochen wurden. Gleichzeitig berichteten einige der Einrichtungen, sich um entsprechende Folgen gesorgt und teilweise zwischen Erfüllung des Gesetzes und dem Auftreten von Versorgungsengpässen abgewogen zu haben (Zitat H3, Tab. 3).

##### Unklarheiten bei der Umsetzung.

Die Mehrheit der Einrichtungen und alle GÄ merkten an, dass Details der Impfpflicht unklar blieben und rechtliche Unsicherheiten bestanden (Zitat H4, Tab. 3). Da die GÄ die Kommunikation und Unterstützung durch bspw. die Landesgesundheitsministerien als unzureichend erlebten, konnten sie sich in ihren Entscheidungen nicht absichern und befürchteten Konsequenzen bei „falschen“ Entscheidungen. Dies schwächte die Handlungssicherheit der GÄ (Zitat H5, Tab. 3).

##### Fehlender Überblick über Einrichtungen.

Die GÄ berichteten, nur teilweise zu überblicken, welche Einrichtungen meldepflichtig waren. Einzelne GÄ sahen die Verantwortung zur Meldung bei den Einrichtungen. Andere behalfen sich, indem sie Listen der meldepflichtigen Einrichtungen zusammenstellten, diese mit den meldenden Einrichtungen abglichen und gegebenenfalls nachfragten. Da zudem keine Pflicht zur Nullmeldung bestand, konnte eine fehlende Meldung der Einrichtungen nicht eindeutig interpretiert werden (Zitat H6, Tab. 3). Die Mehrheit der GÄ vermutete, dass große Einrichtungen gemeldet haben, kleinere und mittlere Einrichtungen aber wahrscheinlich nicht vollständig (Zitat H7, Tab. 3).

##### Arbeitsaufwand durch COVID-19-Impfpflicht.

Die Umsetzung der Impfpflicht ging bei der Mehrheit der Einrichtungen sowie bei allen GÄ mit großem Arbeitsaufwand einher (Zitat H8, H9, Tab. 3). Die GÄ waren insbesondere damit ausgelastet, neue Verwaltungsstrukturen zur Erfassung und Bearbeitung der Meldungen zu schaffen und Rückfragen der Einrichtungen bzw. Betroffenen zu beantworten. Bei den Einrichtungen kam die Kommunikation mit Mitarbeitenden zur Impfpflicht und der Umgang mit impfkritischen Personen dazu. Beides wurde als sehr arbeitsintensiv und belastend beschrieben. Eine fehlende digitale Ausstattung erhöhte den Aufwand in einigen Einrichtungen zusätzlich.

##### Emotionale Belastungen und Spannungen im Umgang mit (ungeimpften) Beschäftigten.

Der Umgang mit Beschäftigten allgemein und insbesondere mit Ungeimpften wurde von allen Einrichtungen und GÄ als emotional belastend wahrgenommen. Dabei war es eine besondere Herausforderung, die Notwendigkeit der Impfpflicht zu kommunizieren (Zitat H10, H11, Tab. 3). Bei den Mitarbeitenden einiger Einrichtungen kam zudem das Gefühl der Ungleichbehandlung gegenüber der Allgemeinbevölkerung auf. Sie hatten den Eindruck, dass die Last der Pandemie auf sie abgewälzt würde (Zitat H12, Tab. 3). Zusätzlich kam es in einzelnen Einrichtungen zu Anfeindungen bzw. Schuldzuweisungen von Geimpften gegenüber Ungeimpften (Zitat H13, H14, Tab. 3).

## Diskussion

Diese Studie untersucht erstmals die praktische Umsetzung der Masernimpfpflicht und der COVID-19-Impfpflicht in Deutschland und liefert darüber hinaus wichtige Erkenntnisse für die Evaluation von Impfpflichten im Allgemeinen. Beide hier untersuchten Impfpflichten weisen viele Parallelen hinsichtlich der erlebten Herausforderungen auf, die im Folgenden integriert diskutiert werden.

### Charakteristiken der Impfpflichten im Kontext

Die Umsetzung der Masernimpfpflicht wurde direkt nach ihrer Einführung wegen der aufkommenden COVID-19-Pandemie depriorisiert. Die GÄ orientierten sich bei ihren Umsetzungsplänen an der Übergangsfrist, die als Auftakt für die Verhängung von Sanktionen verstanden und aufgrund der Pandemie 2‑mal verschoben wurde. Die Umsetzung war zwar bis zum Zeitpunkt der Interviews stark verzögert und Sanktionen kamen kaum zum Einsatz, aber als zeitlich unbegrenzte Impfpflicht besteht nun die Chance, diese erneut anzugehen.

Die einrichtungsbezogene COVID-19-Impfpflicht musste unter hohem Zeitdruck in wenigen Monaten umgesetzt werden und galt in einem Zeitraum, als sich mit der Omikron-Welle die epidemiologische Lage und die Wirksamkeit der Impfstoffe änderten. Unter der Omikron-Variante konnte durch Impfung eine Virusübertragung zwar immer noch reduziert werden, aber nicht mehr im gleichen Maße wie noch während der Delta-Variante [11]. Infolge wandelte sich die Pandemiepolitik und andere Maßnahmen wurden beendet [12–15]. In den Interviews zeigte sich deutlich, dass die COVID-19-Impfpflicht in diesem begrenzten Zeitrahmen nicht konsequent umsetzbar war und ihre Akzeptanz abnahm. Ein zentrales Problem war, dass die im Gesetz vorgesehenen Sanktionen nicht rechtzeitig umgesetzt werden konnten, da die zeitlichen Abläufe der Verwaltungsprozesse zu langwierig waren. Dies wirft die Frage auf, wie eine Impfpflicht trotz kurzer Laufzeit sinnvoll implementiert werden kann. Dem gegenüber stehen Ergebnisse aus nicht-repräsentativen Befragungen von Langzeitpflegeeinrichtungen, die zeigen, dass die Impfquoten (Grundimmunisierung) zwischen November 2021 und März 2022 kontinuierlich stiegen (von 81,3 % auf 92,5 %). Dies kann als Hinweis darauf interpretiert werden, dass die Anfang Dezember 2021 in Kraft getretene COVID-19-Impfpflicht trotz ihrer kurzen Laufzeit zu einer Steigerung der Impfquote beigetragen hat [16].

### Zielkonflikte

Die Umsetzung der Masernimpfpflicht führte zu einem Zielkonflikt: Der Infektionsschutz vor Masern stand hier möglichen sozialen Härten gegenüber. GÄ mussten abwägen, was Betretungsverbote und Bußgelder für Personen bedeuten, für die ein Kindergartenplatz eine elementare Bildungschance darstellt, die keine Alternative zur Gemeinschaftsunterkunft haben oder die ein Bußgeld nicht zahlen können. Hier liefert die Studie erste Hinweise dafür, dass die Masernimpfpflicht soziale Ungleichheiten verstärken könnte, wie dies auch bei Impfpflichten in anderen Ländern beobachtet wurde [4, 6, 17].

Die COVID-19-Impfpflicht trat während einer emotional aufgeladenen Public-Health-Krise in Kraft [18]. Vor diesem Hintergrund standen die Einrichtungen auch hier vor einem Zielkonflikt: Sie wollten einerseits die COVID-19-Impfpflicht durchsetzen und damit Infektionen verhindern, sorgten sich aber andererseits um Eigenkündigungen und daraus resultierende Personalengpässe. Hinzu kam ein Gefühl der Ungleichbehandlung der Mitarbeitenden gegenüber der Allgemeinbevölkerung, teilweise kam es außerdem zu Konflikten zwischen geimpftem und ungeimpftem Personal. Die Interviewdaten unterstreichen die psychische Belastung durch die Pandemie [19, 20], insbesondere im Gesundheitssektor [21] und durch die Polarisierung von Gruppen aufgrund ihres Impfstatus [22, 23].

### Rechtlicher und organisatorischer Rahmen

Für beide Impfpflichten bestanden Unklarheiten bei der Umsetzung, die die Umsetzenden insbesondere bei rechtlichen Belangen in ihrer Handlungssicherheit schwächten. Seitens der Länder und des Bundes gab es nach Aussagen der Teilnehmenden nur wenig Unterstützung. So konnten sich die GÄ in ihren Entscheidungen nicht absichern und fürchteten mögliche rechtliche Konsequenzen. Durch uneinheitliche Vorgaben entstanden lokal unterschiedliche Lösungen, was die Akzeptanz der Maßnahmen bei den Bürger:innen mindern könnte [24]. Landes- bzw. bundesweit einheitliche Vorgaben sowie zentrale Anlaufstellen für Rückfragen würden zu klaren, einheitlichen Abläufen beitragen und den Umsetzenden einen sicheren Handlungsrahmen bieten. Dies würde auch im Umgang mit Personen helfen, die Gefälligkeitsatteste und Verzögerungstaktiken nutzen, um sich dem Gesetz zu entziehen.

Zuletzt war der fehlende Überblick zu den meldepflichtigen Einrichtungen und eine fehlende Verpflichtung zur Nullmeldung für GÄ bei beiden Impfpflichten ein wesentliches Problem. Eine verlässliche Datengrundlage ist jedoch Voraussetzung für standardisierte Abläufe. Für das MSG ergab sich als zusätzliche Schwierigkeit, dass Einrichtungsleitungen als medizinische Laien mit der Kontrolle von Nachweisen überfordert waren. Hier fehlte es an Unterstützung bei der Überprüfung oder an einheitlichen Vorgaben, Atteste grundsätzlich an die GÄ weiterzuleiten.

### Stärken und Schwächen der Studie

Bei den Teilnehmenden wurde bewusst auf die Diversität von Perspektiven geachtet. So waren Bundesländer mit hohen und niedrigen Impfquoten vertreten, unter den Teilnehmenden fanden sich vielfältige Professionen und Einrichtungstypen/GÄ. Der Zeitpunkt der Interviews lag für die COVID-19-Impfpflicht während ihrer Laufzeit und war damit angemessen. Bei der Masernimpfpflicht lief die Übergangsfrist, die oft erst als Auftakt für die Umsetzung des MSG gesehen wurde, erst kurz vor dem Befragungszeitraum ab. Sanktionen, die danach ausgesprochen wurden, wurden durch die Interviews nicht abgedeckt.

Der Leitfaden wurde sorgfältig entwickelt und pilotiert, 2 Interviewer:innen mit mehrjähriger Erfahrung in der qualitativen Forschung wurden explizit für die Studie geschult. Die 1:1-Interviewsituation mit einem externen Auftragnehmer schuf eine Gesprächssituation, in der sich die Teilnehmenden möglichst frei zu ihren Erfahrungen äußern konnten.

Die Daten wurden jeweils von einer Person kodiert und paraphrasiert, eine Interkoderreliabilität wurde somit nicht bestimmt. Die Datenerhebung und -analyse erfolgten systematisiert mittels Framework-Analyse. Sie wurde von einer Person durchgeführt und Zwischenergebnisse mehrfach im Kernforschungsteam besprochen [25]. Diese Herangehensweise an Datenanalyse und -interpretation schafft Vertrauen in die Belastbarkeit der Ergebnisse. Für die Masernimpfpflicht zeigen sich zudem große Ähnlichkeiten bei den Ergebnissen im Vergleich zu einer früheren Befragung [26].

## Fazit

Die vorliegende Studie zielte darauf ab, die Umsetzung der Masernimpfpflicht und der COVID-19-Impfpflicht in Deutschland sowie die damit verbundenen Herausforderungen zu untersuchen. Dabei zeigte sich, dass beide Impfpflichten, obwohl sie streng konzipiert waren [17], kaum bzw. im Fall der Masernimpfpflicht bislang wenig Wirkung entfalten konnten. Für das MSG lag das am Kontext der Einführung (COVID-19-Pandemie), bei der COVID-19-Impfpflicht an der Konzeption (zeitliche Begrenzung bei dynamischer Evidenzlage). Die gemeinsamen Herausforderungen lagen für beide Impfpflichten vor allem in verschiedenen Zielkonflikten und dem rechtlichen und organisatorischen Rahmen. Insbesondere für die Masernimpfpflicht ist jetzt der richtige Zeitpunkt, um diese erneut in den Fokus zu rücken: Viele GÄ hatten die Übergangsfrist bis zum 31.07.2022 abgewartet.

Letztlich bleibt die Entscheidung für oder gegen eine Impfpflicht eine politische. Die Studie liefert jedoch wertvolle Hinweise darauf, welche Faktoren für eine erfolgreiche Umsetzung von Impflichten in Deutschland und potenziell auch in anderen Ländern verbessert werden können.

### Infobox 1

**Das Masernschutzgesetz (MSG)**

- Inkrafttreten: 01.03.2020
- Ziel: Erhöhung der Impfquote, um Zahl der Masernfälle zu reduzieren und somit die Gesundheit der Bevölkerung zu schützen
- Gesetzliche Grundlage: § 20 des Infektionsschutzgesetzes (IfSG)
- Dauer: unbefristet
- Art: einrichtungsbezogene Nachweispflicht
- Betroffene/Zielgruppe: Kinder und Jugendliche, die z. B. in Kindertagesstätten, Schulen oder Gemeinschaftseinrichtungen betreut werden; nach 1970 geborene Beschäftigte in diesen sowie medizinischen Einrichtungen (vollständige Liste siehe § 23 Absatz 3 Satz 1 IfSG)
- Nachweis: über eine (Kinder < 24 Monate) bzw. 2 (Kinder ≥ 24 Monate) Masernimpfungen oder eine labordiagnostisch bestätigte Maserninfektion
- Ausnahmeregelung: medizinische Kontraindikation
- Sanktionen: Buß- oder Zwangsgelder, Ausschluss von der Betreuung, Verbot der Einstellung
- Übergangsfrist für bereits Betreute/Beschäftigte: 31.07.2022 (initial: 31.07.2021)

**Die COVID-19-Impfpflicht**

- Inkrafttreten: 10.12.2021
- Ziel: Schutz besonders gefährdeter Personen in Gesundheitseinrichtungen vor Infektion und schweren Krankheitsverläufen, besondere Verantwortung des dort arbeitenden Personals durch engen Kontakt zu diesen Personen
- Gesetzliche Grundlage: § 20a des IfSG
- Dauer: befristet (bis zum 01.01.2023)
- Art: einrichtungsbezogene Nachweispflicht
- Betroffene/Zielgruppe: Beschäftigte in Gesundheitseinrichtungen (vollständige Liste siehe § 20a IfSG)
- Nachweis: über COVID-19-Impfung oder eine Genesung („Genesenennachweis“)
- Ausnahmeregelung: medizinische Kontraindikation
- Sanktionen: Bußgelder, Betretungs- bzw. Tätigkeitsverbot, Verbot der Einstellung
- Übergangsfrist: 15.03.2022

## Supplementary Information

Interviewleitfaden zur Masernimpfpflicht (Gesundheitsämter)
Interviewleitfaden zur COVID-19-Impfpflicht (Gesundheitsämter, Arztpraxen, Krankenhäuser, Pflegeeinrichtungen)

## Footnotes

1 „Die Elimination der Masern … ist erreicht, wenn eine endemische Transmission von Masernviren … in einem Land über mindestens 36 Monate ausgeschlossen werden konnte. Eine endemische Transmission liegt definitionsgemäß vor, wenn die durch eine Masernvirusvariante ausgelöste Transmissionskette in Deutschland über 12 Monate oder länger nachgewiesen wird“ [1].

2 NSK und JN verfügen über umfangreiche Expertise im Bereich der Impfakzeptanz, insbesondere in der quantitativen Forschung und der Evaluation von Interventionen zur Impfquotensteigerung. Beide haben bereits Erfahrung in der qualitativen Forschung.
