# Supplementary material for: Evaluation of the implementation of the measles and COVID-19 vaccination mandates by public health authorities and health facilities: 2 case studies
Source: Bundesgesundheitsblatt Gesundheitsforschung Gesundheitsschutz. 2025 Mar 25;68(4):432–43. [Article in German] doi: 10.1007/s00103-025-04027-3 (PMC11950079; doi:10.1007/s00103-025-04027-3)
Supplement: Supplementary file 1 — Interviewleitfaden zur Masernimpfpflicht (Gesundheitsämter) Interviewleitfaden zur COVID-19-Impfpflicht (Gesundheitsämter, Arztpraxen, Krankenhäuser, Pflegeeinrichtungen) [file 103_2025_4027_MOESM1_ESM.pdf]

Onlinematerial

zur Publikation: „Evaluation der Umsetzung der COVID-19- und Masern-Impfpflichten in Gesundheitsämtern und Gesundheitseinrichtungen: Zwei Fallstudien“ (Schmid-Küpke et al., 2025)

1. Leitfaden zur Masernimpfpflicht

Gesundheitsämter

Begrüßung

Guten Tag und herzlich willkommen! Vielen Dank, dass Sie sich dazu bereit erklärt haben, an diesem Interview zur Masern-Impfpflicht teilzunehmen.

Bevor wir loslegen ein paar erläuternde Worte:

Wir möchten heute von Ihnen erfahren, wie Sie in Ihrem Gesundheitsamt die Masern-Impfpflicht umgesetzt haben und welche Herausforderungen Sie bei der Umsetzung der Impfpflicht möglicherweise zu meistern hatten. Dafür führen wir Interviews mit ausgewählten Expert*innen wie Ihnen durch, die bei der Umsetzung der Masern-Impfpflicht aktiv beteiligt sind. Da wir an Ihrer Meinung und Ihren Erfahrungen interessiert sind, gibt es bei den Antworten auf die Fragen kein richtig oder falsch und wir bitten Sie darum, frei heraus zu reden.

Wir werden dieses Gespräch aufnehmen und nutzen auch dafür Webex. Mit Hilfe der Aufnahme werden wir das Interview transkribieren und hinterher auswerten. Bei der Transkription werden alle personenbezogenen Daten, die eventuell im Gespräch geäußert werden, anonymisiert, sodass keine Rückschlüsse auf Sie oder andere Personen möglich sind. Alle anderen personenbezogenen Daten (u.a. Ihre Kontaktdaten) und auch die Audioaufnahmen werden nach der Transkription und Qualitätssicherung der Transkripte gelöscht, nicht an unbefugte Dritte weitergegeben, nicht vervielfältigt oder in dieser Form veröffentlicht.

Ihre Teilnahme an dem Interview ist absolut freiwillig und Sie können Ihre Einwilligung zur Teilnahme jederzeit widerrufen. Haben Sie vor Beginn der Aufnahme noch Fragen?

**Wenn keine Rückfragen:* Dann würde ich die Aufnahme jetzt starten* *(Aufnahme starten)*

Intro

Zu Beginn würde uns erstmal interessieren, welche Rolle Sie in Ihrem Gesundheitsamt haben, wie Sie in die Umsetzung der Impfpflicht eingebunden sind und ob auch noch weitere Personen bei Ihnen im Gesundheitsamt da mitarbeiten. Fangen wir doch an mit Ihrer Rolle im Gesundheitsamt.

1. **Prozess** → Wie wurde die Masern-Impfpflicht durch Gesundheitsämter umgesetzt?

Zunächst würde uns interessieren, wie das bei Ihnen mit der Masern-Impfpflicht so abgelaufen ist.

- Können Sie sich erinnern wie (und wann?) Sie von der Impfpflicht erfahren haben?
  - Gab es am Anfang Unterstützung vom Bundesgesundheitsministerium oder vom Land und wie sah die aus? Und wie war das im Zeitverlauf (wenn Sie jetzt an die erste Zeit nach dem In-Kraft-Treten des Gesetzes denken und an die letzten Monate)?
  - Haben Sie sich zur Umsetzung der Impfpflicht mit anderen Gesundheitsämtern oder auf Landesebene ausgetauscht? Worüber?
  - Haben Sie mit den Einrichtungen Kontakt aufgenommen? Wann ist das geschehen?
  - Hatten Sie Fragen zur Impfpflicht? Wem konnten Sie diese stellen?

- Haben Sie sich irgendwie auf die Impfpflicht vorbereitet?
  - Wie? Wann ist das passiert?
  - Haben Sie sich nochmal neu oder anders vorbereitet, jetzt, wo die Übergangsfrist abgelaufen ist?

- **Wie haben Sie die Masern-Impfpflicht konkret bei sich vor Ort umgesetzt? Erzählen Sie doch mal.**
  - **Woher wissen Sie, wer noch einen Masernschutz nachweisen muss? Wie behalten Sie da den Überblick? Wie haben Sie das mit Personen gehandhabt, die noch bis zur Übergangsfrist Zeit hatten, einen Masernschutz nachzuweisen?**
  - **Haben mittlerweile alle Einrichtungen (die Nachweise zum Masernschutz) an Sie gemeldet?**
  - **Wurden Meldungen von Personen, die keinen Masernschutz nachweisen können, schon alle bearbeitet?** **Hier ggf. gern auch die verschiedenen Einrichtungen direkt ansprechen, i.S. von „Was ist mit Kita, Schule, Sammelunterkünften, Arztpraxen, Krankenhäusern?“, falls das sehr einseitig nur in Bezug auf eine Einrichtung berichtet wird*
  - **Was wurde denn gemacht, wenn jemand keinen Masernschutz nachweisen konnte?**
    - **Gab es Gespräche, kam es zu Geldbußen, Betretungsverboten?**
  - **Welche Priorität wurde der Umsetzung der Impfpflicht bei Ihnen im Gesundheitsamt eingeräumt?**

2. **Herausforderungen** → Welche Herausforderungen gibt es in der Umsetzung der Masern-Impfpflicht?

Vielen Dank, das waren ja schon mal sehr spannende Einblicke! Als nächstes interessiert uns, ob Sie Herausforderungen im Zusammenhang mit der Masern-Impfpflicht erlebt haben…

- Dazu erstmal ganz offen gefragt: Welche Herausforderungen rund um die Impflicht kommen Ihnen da als erstes in den Sinn? Welche haben Sie selbst erlebt? **Hier flexibel handhaben: wenn weiter oben schon die Herausforderungen zu diesen Prompts ausführlich besprochen wurden, muss es hier nicht mehr explizit gefragt werden. Bestenfalls können wir das ein stückweit trennen: oben neutrale Beschreibung, hier dann mehr Meinung und Erfahrung**
    - **Was würden Sie sagen, welches war die größte Herausforderung?**
    - Wie haben Sie die Unterstützung und Anleitung durch den Bund oder das Land empfunden? Hat sich das über die Zeit verändert?
    - **Wir denken hier auch an die Übergangsfrist, die ja erst kürzlich abgelaufen ist. Sind dadurch neue Herausforderungen auf Sie zugekommen? Welche?**
    - Gibt es Stand heute noch Unsicherheiten bei der Umsetzung des Gesetzes, bspw. rechtliche Unsicherheiten oder Unsicherheiten bei Kontraindikationen?
      **Mit Kontraindikationen ist an dieser Stelle gemeint, dass die Leute bspw. Multiple Sklerose haben und wahrhaftig unsicher sind, ob eine Impfung für sie sicher/geeignet ist. Es handelt sich dabei meistens um unnötige Sorgen, die die Leute sich vor der Impfung machen, aber hier könnte echte Unsicherheit bestehen. Das Gesundheitsamt könnte hier selbst nicht genau wissen, was eine echte Kontraindikation ist und was nicht.*
    - Gab es bei Ihnen im Haus genügend personelle Ressourcen für die Umsetzung? Hätten Sie noch etwas anderes gebraucht?
    - Wie würden Sie die Zusammenarbeit mit den Einrichtungen bewerten? Was war gelungen, was hätte besser laufen können?
    - **Welche Erfahrungen haben Sie mit Personen gemacht, die keinen Nachweis erbringen können?**
        - **Wie war Ihre Erfahrung mit Gefälligkeitsattesten?**
        - **Haben Sie juristische und andere Verzögerungstaktiken miterlebt? Können Sie davon berichten?**
    - **Wir haben von der Befürchtung gehört, dass das tatsächliche Ausmaß impfkritischer Eltern möglicherweise erst nach Ablauf der Übergangsfrist zum Vorschein kommt, weil sich teilweise auf diesen Termin berufen wurde und der Nachweis nicht früher vorgelegt werden wollte. Deckt sich das mit Ihrem Eindruck? Wie war Ihre <u>Erfahrung</u> nach Ablauf der Übergangsfrist?**

- Gut, Sie haben jetzt von Ihren Erfahrungen berichtet, Sie haben ja sicher auch einiges bei der Umsetzung gelernt. Uns würde interessieren, wie Sie mit den von Ihnen genannten Herausforderungen umgegangen sind?
    - Was würden Sie sagen, was waren oder sind aus Ihrer Sicht gute Strategien im Umgang mit … (gewesen)? **Hier konkret nach den Strategien im Umgang mit den Herausforderungen fragen, die im der vorherigen Gespräch besonders prominent/ relevant waren**

- Fehlender Unterstützung durch Bund oder Land
- Gefälligkeitsattesten
- juristischen und andere Verzögerungstaktiken
- impfkritischen Eltern (insbesondere nach der Übergangsfrist)

**Wenn im Gespräch noch weitere Herausforderungen genannt werden, die hier nicht auftauchen, die aber wichtig scheinen, dann gern hier auch nach Strategien fragen.*

Outro

Vielen Dank! Wir sind nun fast am Ende unseres Interviews angekommen. Deshalb möchte ich Ihnen jetzt die Gelegenheit geben:

- Wie bewerten Sie denn selbst den Sinn bzw. die Effektivität der Impfpflicht?
- Liegt Ihnen noch etwas auf dem Herzen, was Sie gerne loswerden möchten? Haben wir etwas Wichtiges nicht angesprochen?

Verabschiedung

Vielen Dank für das interessante Gespräch, für die Einblicke in Ihre Arbeit und Ihre persönlichen Sichtweisen zur Masern-Impfpflicht.

- Gerne lassen wir Ihnen die Ergebnisse der Interviews als Kurzbericht zukommen. Wenn Sie daran interessiert sind, schicken Sie uns bitte einfach eine kurze Mail an xxx@rki.de
- Für die Aufwandsentschädigung…

Wir wünschen Ihnen noch einen schönen Tag!

2. Leitfäden zur COVID-19-Impfpflicht

Gesundheitsämter

Begrüßung

Guten Tag und herzlich willkommen! Vielen Dank, dass Sie sich dazu bereit erklärt haben, an diesem Interview zur (einrichtungsbezogenen) COVID-19 Impfpflicht teilzunehmen.

Bevor wir loslegen ein paar erläuternde Worte:

Wir möchten heute von Ihnen erfahren, wie Sie in Ihrem Gesundheitsamt die COVID-19 Impfpflicht umsetzen, welche Erfahrungen Sie dabei in Bezug auf Sanktionen und entstehende Herausforderungen gemacht haben. Dafür führen wir Interviews mit ausgewählten Expert*innen wie Ihnen durch, die bei der Umsetzung der Impfpflicht aktiv beteiligt sind. Da wir an Ihrer Meinung und Ihren Erfahrungen interessiert sind, gibt es bei den Antworten auf die Fragen kein richtig oder falsch und wir bitten Sie darum, frei zu reden.

Wir werden dieses Interview für die Verarbeitung der gesammelten Daten aufnehmen und nutzen auch dafür Webex. Mit Hilfe der Aufnahme werden wir das Interview transkribieren und hinterher auswerten. Bei der Transkription werden alle personenbezogenen Daten, die eventuell im Interview geäußert werden, anonymisiert, sodass keine Rückschlüsse auf Sie oder andere Personen möglich sind. Alle anderen personenbezogenen Daten (u.a. Ihre Kontaktdaten) und auch die Audioaufnahmen werden nach der Transkription und Qualitätssicherung der Transkripte gelöscht, nicht an unbefugte Dritte weitergegeben, vervielfältigt oder in dieser Form veröffentlicht.

Ihre Teilnahme an dem Interview ist absolut freiwillig und Sie können Ihre Einwilligung zur Teilnahme jederzeit widerrufen. Haben Sie vor Beginn der Aufnahme noch Fragen?

**Wenn keine Rückfragen:* Dann würde ich die Aufnahme jetzt starten* *(Aufnahme starten)*

Intro

Zu Beginn würde uns erstmal Ihre Rolle im Gesundheitsamt interessieren. Bitte beschreiben Sie daher kurz Ihre Funktion, wie Sie in die Umsetzung der Impfpflicht eingebunden sind und ob auch noch weitere Personen bei Ihnen im Gesundheitsamt da mitarbeiten.

---

3. **Prozess** → Wie wird die einrichtungsbezogene COVID-19 Impfpflicht von Einrichtungsleitungen und durch Gesundheitsämter umgesetzt?

Zunächst würde uns interessieren, wie das bei Ihnen mit der Impfpflicht abgelaufen ist.

- Wie (und wann?) haben Sie von der Impfpflicht erfahren? Können Sie sich noch daran erinnern?
  - Haben Sie sich irgendwie auf die Impfpflicht vorbereitet? Wie?
  - Gab es Unterstützung vom Bundesgesundheitsministerium oder von den Gesundheitsministerien des Landes und wie sah diese aus?
  - Wie war der Austausch zur Umsetzung der Impfpflicht mit anderen Gesundheitsämtern auf Landes- oder auf Bundesebene? Gab es einen?
  - Hatten Sie Fragen zur Impfpflicht? Wem konnten Sie diese stellen?

- Sie hatten ja schon angedeutet, dass unterschiedliche Personen in die Umsetzung der Impfpflicht eingebunden sind: Welche Stellen sind denn bei Ihnen konkret mit der Umsetzung der Impfpflicht beschäftigt?
  - **Und welche Stelle übernimmt da welche Aufgaben? (z.B. Verhängung von Sanktionen, …)**
  - Ist die Rollenzuweisung eindeutig? Weiß jeder was er*sie zu tun hat?
    Hier darauf hinweisen, dass es nicht um Namen geht

- Nun wollen wir ein bisschen mehr darüber erfahren, wie Sie die Impfpflicht konkret umgesetzt haben. Erzählen Sie doch mal.
  - **Woher wissen sie, wer eine COVID-19-Impfung nachweisen muss?**
  - **Wie viele Einrichtungen haben mittlerweile an Sie gemeldet? Alle? (Informationen zum COVID-Impfstatus aus allen Einrichtungen)**
  - **Wurden Meldungen von Personen, die keine COVID-19 Impfung nachweisen können, schon alle bearbeitet?**
  - **Was wurde denn gemacht, wenn jemand keine COVID-19-Impfung nachweisen konnte? Gab es Gespräche, kam es zu Geldbußen, Betretungsverboten?**
  - **Wie läuft es ab, nun da seit 01. Oktober nicht mehr zwei, sondern drei Impfungen notwendig sind: finden erneut Kontrollen bei Ihnen statt oder wie gehen Sie damit um?**

---

4. **Herausforderungen** → Welche Herausforderungen gibt es in der Umsetzung der einrichtungsbezogenen COVID-19 Impfpflicht?

Vielen Dank, das waren ja schon mal sehr spannende Einblicke! Als nächstes interessiert uns, ob Sie Herausforderungen im Zusammenhang mit der Impfpflicht erlebt haben…

- Dazu erstmal ganz offen gefragt: Welche Herausforderungen rund um die Impflicht kommen Ihnen da als erstes in den Sinn? Welche haben Sie selbst erlebt?
    - **Was würden Sie sagen, welche war die größte Herausforderung?**
    - Wie haben Sie die Unterstützung durch den Bund und das Land empfunden?
    - Gab es bei Ihnen im Haus genügend personelle Ressourcen für die Umsetzung? Hätten Sie noch etwas anderes gebraucht?
    - Wie würden Sie die Zusammenarbeit mit den Einrichtungen bewerten? (Was war gelungen, was hätte besser laufen können?)
    - **Welche Erfahrungen haben Sie mit Personen gemacht, die keinen Nachweis erbringen können?**
        - **Wie war Ihre Erfahrung mit Gefälligkeitsattesten?**
        - **Haben Sie juristische und andere Verzögerungstaktiken erlebt? Können Sie davon berichten?**

- **Was würden Sie sagen, was waren oder sind aus Ihrer Sicht gute Strategien im Umgang mit … (gewesen)?** **Hier konkret nach den Strategien im Umgang mit den Herausforderungen fragen, die in dem vorherigen Gespräch besonders prominent/ relevant waren**
        - Fehlender Unterstützung durch Bund und Land
        - Gefälligkeitsattesten
        - juristischen und andere Verzögerungstaktiken
        - Personen, die keinen Nachweis erbringen können
        - Fehlenden Ressourcen
        - Situativ Strategien im Umgang mit weiteren Herausforderungen…

---

5. **Weitere Bereiche** → Welche Effekte hat die einrichtungsbezogene COVID-19 Impfpflicht auf andere Bereiche?

Wir haben uns jetzt ausführlich darüber unterhalten, wie die Impfpflicht bei Ihnen im Gesundheitsamt umgesetzt wird, welche Herausforderungen Sie erlebt haben und wie Sie diese bewältigt haben. So eine Impfpflicht kann sich ja auch auf andere Bereiche des Arbeitslebens auswirken.

- Gab es Dinge, die in Ihrem Gesundheitsamt auf Grund der COVID-19-Impfpflicht liegen geblieben sind? Welche sind das?
    - Betrifft das auch Ihre Arbeit?
    - Welche Auswirkungen hatte das, oder könnte das noch haben?
    - **Haben sich Ihre eigentlichen Arbeitsaufgaben mit den neuen Aufgaben zur Impfpflicht verändert?**

Outro

Die Impfpflicht wird ja zum 01.01.2023 voraussichtlich auslaufen.

- Was halten Sie davon?
- Unterstützen Sie das oder nicht?
- Warum?

Vielen Dank! Wir sind nun fast am Ende unserer Diskussionsrunde angekommen. Deshalb möchte ich Ihnen jetzt die Gelegenheit geben:

- Liegt Ihnen noch etwas auf dem Herzen, was Sie gerne loswerden möchten? Haben wir etwas Wichtiges nicht angesprochen?

Verabschiedung

Vielen Dank für das interessante Gespräch, für die Einblicke in Ihre Arbeit und Ihre persönlichen Sichtweisen zur Impfpflicht.

- Gerne lassen wir Ihnen die Ergebnisse des Interviews als Kurzbericht zukommen… Wenn Sie daran interessiert sind, schicken Sie uns bitte einfach eine kurze Mail an xxx@rki.de
- Für die Überweisung der Aufwandsentschädigung…

Wir wünschen Ihnen noch einen schönen Tag!

Begrüßung

Guten Tag und herzlich willkommen! Vielen Dank, dass Sie sich dazu bereit erklärt haben, an diesem Interview zur (einrichtungsbezogenen) COVID-19 Impfpflicht teilzunehmen.

Bevor wir loslegen ein paar erläuternde Worte:

Wir möchten heute von Ihnen erfahren, wie Sie in Ihrer Praxis die COVID-19 Impfpflicht umsetzen, welche Herausforderungen es dabei gab und wie sich die Arbeit bei Ihnen durch die Impfpflicht verändert hat. Dafür führen wir Interviews mit ausgewählten Expert*innen wie Ihnen durch, die bei der Umsetzung der Impfpflicht aktiv beteiligt sind. Da wir an Ihrer Meinung und Ihren Erfahrungen interessiert sind, gibt es bei den Antworten auf die Fragen kein richtig oder falsch und wir bitten Sie darum, frei zu reden.

Wir werden dieses Interview für die Verarbeitung der gesammelten Daten aufnehmen und nutzen auch dafür Webex. Mit Hilfe der Aufnahme werden wir dieses Interview transkribieren und hinterher auswerten. Bei der Transkription werden alle personenbezogenen Daten, die eventuell im Interview geäußert werden, anonymisiert, sodass keine Rückschlüsse auf Sie oder andere Personen möglich sind. Alle anderen personenbezogenen Daten (u.a. Ihre Kontaktdaten) und auch die Audioaufnahmen werden nach der Transkription und Qualitätssicherung der Transkripte gelöscht, nicht an unbefugte Dritte weitergegeben, vervielfältigt oder in dieser Form veröffentlicht.

Ihre Teilnahme an dem Interview ist absolut freiwillig und Sie können Ihre Einwilligung zur Teilnahme jederzeit widerrufen. Haben Sie vor Beginn der Aufnahme noch Fragen?

**Wenn keine Rückfragen:* Dann würde ich die Aufnahme jetzt starten* *(Aufnahme starten)*

Intro

Zu Beginn würde uns erstmal interessieren, welche Rolle Sie in Ihrer Praxis haben, wie Sie in die Umsetzung der Impfpflicht eingebunden sind und ob auch noch weitere Personen bei Ihnen in der Praxis da mitarbeiten.

---

1. **Prozess** → Wie wird die einrichtungsbezogene COVID-19 Impfpflicht von Einrichtungsleitungen und durch Gesundheitsämter umgesetzt?

Zunächst würde uns interessieren, wie das bei Ihnen mit der Impfpflicht abgelaufen ist.

- Wie (und wann?) haben Sie von der Impfpflicht erfahren?
  - Haben Sie sich irgendwie auf die Impfpflicht vorbereitet? Wie?
  - Gab es Unterstützung vom Bundesgesundheitsministerium oder von den Gesundheitsministerien des Landes und wie sah diese aus?
  - **Haben Sie sich zur Umsetzung der Impfpflicht mit anderen Arztpraxen, z.B. bei Konferenzen oder Fachverbänden ausgetauscht? Worum ging es da?**
  - Hatten Sie Fragen zur Impfpflicht? Wem konnten Sie diese stellen?

- Sie hatten ja schon beschrieben, wer an der Umsetzung der Impfpflicht bei Ihnen in der Praxis beteiligt ist: Können Sie da nochmal beschreiben, wer da welche Aufgaben übernimmt? (zum Beispiel die Kontrolle der Nachweise, die Information über die Impfpflicht an die Mitarbeitenden, …)
  - Ist die Rollenzuweisung eindeutig? Weiß jeder was er*sie zu tun hat?
    Hier darauf hinweisen, dass es <u>nicht</u> um Namen geht

- Nun wollen wir ein bisschen mehr darüber erfahren, wie Sie die Impfpflicht konkret bei sich in der Praxis umgesetzt haben. Erzählen Sie doch mal.
  - Wie haben Sie die Impfpflicht an Ihre Mitarbeitenden kommuniziert? (Gab es diesbezüglich spezifische Infoveranstaltungen, Anleitungen?)
  - **Woher wissen Sie, ob Ihre Mitarbeitenden geimpft sind oder nicht?**
    - **Und ab wann wurden die Nachweise kontrolliert?**
  - **Wie und wann wurden Gesundheitsämter mit eingebunden?**
  - **Was haben Sie gemacht, wenn jemand keinen Impfnachweis vorlegen konnte?**
    - **Kam es zu Kündigungen oder wurden andere Maßnahmen getroffen?**
    - **Wie haben Sie zur Impfung motiviert?**
  - **Wie läuft es ab, nun da seit 01. Oktober nicht mehr zwei, sondern drei Impfungen notwendig sind: Finden erneut Kontrollen bei Ihnen statt oder wie gehen Sie damit um?**

---

2. **Herausforderungen** → Welche Herausforderungen gibt es in der Umsetzung der einrichtungsbezogenen COVID-19 Impfpflicht?

Vielen Dank, das waren ja schon mal sehr spannende Einblicke! Als nächstes interessiert uns, ob Sie Herausforderungen im Zusammenhang mit der Impfpflicht erlebt haben…

- Dazu erstmal ganz offen gefragt: Welche Herausforderungen rund um die Impflicht kommen Ihnen da als erstes in den Sinn? Welche haben Sie selbst erlebt?
  - **Was würden Sie sagen, welche war die größte Herausforderung?**
  - Wie haben Sie die Unterstützung durch den Bund, das Land und das Gesundheitsamt empfunden?
  - Gab es bei Ihnen in der Praxis genügend personelle Ressourcen für die Umsetzung? Hätten Sie noch etwas anderes gebraucht?
  - **Welche Erfahrungen haben Sie mit Mitarbeitenden gemacht, die keinen Nachweis erbringen können?**
    - **Wie war Ihre Erfahrung mit Gefälligkeitsattesten oder Verzögerungstaktiken? Können Sie davon berichten?**

- **Was würden Sie sagen, was waren oder sind aus Ihrer Sicht gute Strategien im Umgang mit … (gewesen)?** **Hier konkret nach den Strategien im Umgang mit den Herausforderungen fragen, die im vorherigen Gespräch besonders prominent/ relevant waren**
  - Fehlender Unterstützung durch Bund/Land
  - Gefälligkeitsattesten und Verzögerungstaktiken
  - Impfkritischen oder verärgerten Mitarbeitenden (insbesondere nach der Übergangsfrist)
  - Fehlenden Ressourcen
  - Situativ Strategien im Umgang mit weiteren Herausforderungen…

---

3. **Weitere Bereiche** → Welche Effekte hat die einrichtungsbezogene COVID-19 Impfpflicht auf andere Bereiche?

Wir haben uns jetzt ausführlich darüber unterhalten, wie die Impfpflicht bei Ihnen in der Praxis abläuft und umgesetzt wird, welche Herausforderungen Sie erlebt haben und wie Sie diese bewältigt haben. So eine Impfpflicht kann sich ja auch auf andere Bereiche des Arbeitslebens auswirken.

- Hat sich aus Ihrer Sicht seit der Einführung der Impfpflicht, also seit März dieses Jahres, etwas in Ihrer Praxis verändert? Wir denken da an das Miteinander. Können Sie das beschreiben?
  - Wie gehen geimpfte und ungeimpfte Mitarbeitende miteinander um?
  - **Was haben Sie beobachtet, wie haben die bereits geimpften Mitarbeitenden auf die Einführung der Impfpflicht reagiert?**
  - **Wie haben ungeimpfte Mitarbeitende auf die Einführung der Impfpflicht reagiert?**

- War die Impfpflicht auch bei Ihren Patient:innen ein Thema? Inwiefern?
  - Haben Patient:innen sich erkundigt, ob die Mitarbeitenden geimpft sind?
  - **Und für Sie selbst gesprochen, ist der Schutz der Patient:innen ein guter Grund für eine Impfpflicht oder eher unnötig, warum**?

Outro

Die Impfpflicht wird ja zum 01.01.2023 voraussichtlich auslaufen.

- Was halten Sie davon?
- Unterstützen Sie das oder nicht?
- Warum?

Vielen Dank! Wir sind nun fast am Ende unseres Interviews angekommen. Deshalb möchte ich Ihnen jetzt die Gelegenheit geben:

- Liegt Ihnen noch etwas auf dem Herzen, was Sie gerne loswerden möchten? Haben wir etwas Wichtiges nicht angesprochen?

Verabschiedung

Vielen Dank für das interessante Gespräch, für die Einblicke in Ihre Arbeit und Ihre persönlichen Sichtweisen zur Impfpflicht.

- Gerne lassen wir Ihnen die Ergebnisse der Interviews als Kurzbericht zukommen… Wenn Sie daran interessiert sind, schicken Sie uns bitte einfach eine kurze Mail an xxx@rki.de
- Für die Überweisung der Aufwandsentschädigung…

Wir wünschen Ihnen noch einen schönen Tag!

Begrüßung

Guten Tag und herzlich willkommen! Vielen Dank, dass Sie sich dazu bereit erklärt haben, an diesem Interview zur (einrichtungsbezogenen) COVID-19 Impfpflicht teilzunehmen.

Bevor wir loslegen ein paar erläuternde Worte:

Wir möchten heute von Ihnen erfahren, wie Sie in Ihrem Krankenhaus die COVID-19 Impfpflicht umsetzen, welche Herausforderungen es dabei gab und wie sich die Arbeit bei Ihnen durch die Impfpflicht verändert hat. Dafür führen wir Interviews mit ausgewählten Expert*innen wie Ihnen durch, die bei der Umsetzung der Impfpflicht aktiv beteiligt sind. Da wir an Ihrer Meinung und Ihren Erfahrungen interessiert sind, gibt es bei den Antworten auf die Fragen kein richtig oder falsch und wir bitten Sie darum, frei zu reden.

Wir werden dieses Interview für die Verarbeitung der gesammelten Daten aufnehmen und nutzen auch dafür Webex. Mit Hilfe der Aufnahme werden wir das Interview transkribieren und hinterher auswerten. Bei der Transkription werden alle personenbezogenen Daten, die eventuell im Interview geäußert werden, anonymisiert, sodass keine Rückschlüsse auf Sie oder andere Personen möglich sind. Alle anderen personenbezogenen Daten (u.a. Ihre Kontaktdaten) und auch die Audioaufnahmen werden nach der Transkription und Qualitätssicherung der Transkripte gelöscht, nicht an unbefugte Dritte weitergegeben, vervielfältigt oder in dieser Form veröffentlicht.

Ihre Teilnahme an dem Interview ist absolut freiwillig und Sie können Ihre Einwilligung zur Teilnahme jederzeit widerrufen. Haben Sie vor Beginn der Aufnahme noch Fragen?

**Wenn keine Rückfragen:* Dann würde ich die Aufnahme jetzt starten* *(Aufnahme starten)*

Intro

Zu Beginn würde uns erstmal interessieren, welche Rolle Sie in Ihrem Krankenhaus haben, wie Sie in die Umsetzung der Impfpflicht eingebunden sind und ob auch noch weitere Personen bei Ihnen im Krankenhaus da mitarbeiten.

---

1. **Prozess** → Wie wird die einrichtungsbezogene COVID-19 Impfpflicht von Einrichtungsleitungen und durch Gesundheitsämter umgesetzt?

Zunächst würde uns interessieren, wie das bei Ihnen mit der Impfpflicht abgelaufen ist.

- Wie (und wann?) haben Sie von der Impfpflicht erfahren? Können Sie sich noch daran erinnern?
  - Haben Sie sich irgendwie auf die Impfpflicht vorbereitet? Wie?
  - Gab es Unterstützung vom Bundesgesundheitsministerium oder von den Gesundheitsministerien des Landes und wie sah diese aus?
  - Haben Sie sich zur Umsetzung der Impfpflicht mit anderen Krankenhäusern oder auf Landesebene ausgetauscht?
  - Hatten Sie Fragen zur Impfpflicht? Wem konnten Sie diese stellen?

- Sie hatten ja schon angedeutet, dass unterschiedliche Personen in die Umsetzung der Impfpflicht eingebunden sind: Welche Stellen sind denn bei Ihnen konkret mit der Umsetzung der Impfpflicht beschäftigt?
  - **Und welche Stelle übernimmt da welche Aufgaben? (zum Beispiel die Kontrolle der Nachweise, die Information über die Impfpflicht an die Mitarbeitenden, …)**
  - Ist die Rollenzuweisung eindeutig? Weiß jeder was er*sie zu tun hat?
    Hier darauf hinweisen, dass es nicht um Namen geht

- Nun wollen wir ein bisschen mehr darüber erfahren, wie Sie die Impfpflicht konkret bei sich im Krankenhaus umgesetzt haben. Erzählen Sie doch mal.
  - Wie haben Sie die Impfpflicht im Haus an die Mitarbeitenden kommuniziert? (Gab es diesbezüglich spezifische Infoveranstaltungen, Anleitungen oder interne Kampagnen?)
  - **Woher wissen Sie, ob die Mitarbeitenden geimpft sind oder nicht?**
    - **Und ab wann wurden die Nachweise kontrolliert?**
  - **Wie und wann wurden Gesundheitsämter mit eingebunden?**
  - **Was wurde denn gemacht, wenn jemand keinen Impfnachweis vorlegen konnte?**
    - **Kam es zu Kündigungen, wurden Mitarbeitende versetzt oder welche anderen Maßnahmen wurden getroffen?**
    - **Wie haben Sie zur Impfung motiviert?**

- **Wie läuft es ab, nun da seit 01. Oktober nicht mehr zwei, sondern drei Impfungen notwendig sind: finden erneut Kontrollen bei Ihnen statt oder wie gehen Sie damit um?**

---

2. **Herausforderungen** → Welche Herausforderungen gibt es in der Umsetzung der einrichtungsbezogenen COVID-19 Impfpflicht?

Vielen Dank, das waren ja schon mal sehr spannende Einblicke! Als nächstes interessiert uns, ob Sie Herausforderungen im Zusammenhang mit der Impfpflicht erlebt haben…

- Dazu erstmal ganz offen gefragt: Welche Herausforderungen rund um die Impflicht kommen Ihnen da als erstes in den Sinn? Welche haben Sie selbst erlebt?
  - **Was würden Sie sagen, welche war die größte Herausforderung?**
  - Wie haben Sie die Unterstützung durch den Bund, das Land und das Gesundheitsamt empfunden?
  - Gab es bei Ihnen im Haus genügend personelle Ressourcen für die Umsetzung? Hätten Sie noch etwas anderes gebraucht?
  - Und wie würden Sie die Kommunikation zur Impfpflicht an die Mitarbeitenden bewerten? War sie gelungen oder wie würden Sie diese verbessern?
  - **Welche Erfahrungen haben Sie mit Mitarbeitenden gemacht, die keinen Nachweis erbringen können?**
    - **Wie war Ihre Erfahrung mit Gefälligkeitsattesten oder Verzögerungstaktiken? Können Sie davon berichten?**

- **Was würden Sie sagen, was waren oder sind aus Ihrer Sicht gute Strategien im Umgang mit … (gewesen)?** **Hier konkret nach den Strategien im Umgang mit den Herausforderungen fragen, die im vorherigen Gespräch besonders prominent/ relevant waren**
  - Fehlender Unterstützung durch Bund/ Land
  - Gefälligkeitsattesten und Verzögerungstaktiken
  - Impfkritischen oder verärgerten Mitarbeitenden (insbesondere nach der Übergangsfrist)
  - Fehlenden Ressourcen
  - Situativ Strategien im Umgang mit weiteren Herausforderungen…

---

3. **Weitere Bereiche** → Welche Effekte hat die einrichtungsbezogene COVID-19 Impfpflicht auf andere Bereiche?

Wir haben uns jetzt ausführlich darüber unterhalten, wie die Impfpflicht bei Ihnen im Krankenhaus abläuft und umgesetzt wird, welche Herausforderungen Sie erlebt haben und wie Sie diese bewältigt haben. So eine Impfpflicht kann sich ja auch auf andere Bereiche des Arbeitslebens auswirken.

- Hat sich aus Ihrer Sicht seit der Einführung der Impfpflicht, also seit März dieses Jahres, etwas in Ihrem Krankenhaus verändert? Wir denken da an das Miteinander. Können Sie das beschreiben?

- Wie gehen geimpfte und ungeimpfte Mitarbeitende miteinander um?
- **Was haben Sie beobachtet, wie haben die bereits geimpften Mitarbeitenden auf die Einführung der Impfpflicht reagiert?**
- **Wie haben ungeimpfte Mitarbeitende auf die Einführung der Impfpflicht reagiert?**

- Gab es Dinge, die in Ihrem Krankenhaus auf Grund der Impfpflicht liegen geblieben sind? Welche sind das?
  - Betrifft das auch Ihre Arbeit?
  - Welche Auswirkungen hatte das oder könnte das noch haben?
  - Haben sich Ihre eigentlichen Arbeitsaufgaben mit den neuen Aufgaben zur Impfpflicht verändert?

- War die Impfpflicht auch bei Ihren Patient:innen ein Thema? Inwiefern?
  - Haben Patient:innen sich erkundigt, ob die Mitarbeitenden geimpft sind?
  - **Und für Sie selbst gesprochen, ist der Schutz der Patient:innen ein guter Grund für eine Impfpflicht oder eher unnötig, warum?**

Outro

Die Impfpflicht wird ja zum 01.01.2023 voraussichtlich auslaufen.

- Was halten Sie davon?
- Unterstützen Sie das oder nicht?
- Warum?

Vielen Dank! Wir sind nun fast am Ende unserer Diskussionsrunde angekommen. Deshalb möchte ich Ihnen jetzt die Gelegenheit geben:

- Liegt Ihnen noch etwas auf dem Herzen, was Sie gerne loswerden möchten? Haben wir etwas Wichtiges nicht angesprochen?

Verabschiedung

Vielen Dank für das interessante Gespräch, für die Einblicke in Ihre Arbeit und Ihre persönlichen Sichtweisen zur Impfpflicht.

- Gerne lassen wir Ihnen die Ergebnisse der Interviews als Kurzbericht zukommen… Wenn Sie daran interessiert sind, schicken Sie uns bitte einfach eine kurze Mail an xxx@rki.de
- Für die Überweisung der Aufwandsentschädigung…

Wir wünschen Ihnen noch einen schönen Tag!

Begrüßung

Guten Tag und herzlich willkommen! Vielen Dank, dass Sie sich dazu bereit erklärt haben, an diesem Interview zur (einrichtungsbezogenen) COVID-19 Impfpflicht teilzunehmen.

Bevor wir loslegen ein paar erläuternde Worte:

Wir möchten heute von Ihnen erfahren, wie Sie in Ihrer Einrichtung die COVID-19 Impfpflicht umsetzen, welche Herausforderungen es dabei gab und wie sich die Arbeit bei Ihnen durch die Impfpflicht verändert hat. Dafür führen wir Interviews mit ausgewählten Expert*innen wie Ihnen durch, die bei der Umsetzung der Impfpflicht aktiv beteiligt sind. Da wir an Ihrer Meinung und Ihren Erfahrungen interessiert sind, gibt es bei den Antworten auf die Fragen kein richtig oder falsch und wir bitten Sie darum, frei zu reden.

Wir werden dieses Interview für die Verarbeitung der gesammelten Daten aufnehmen und nutzen auch dafür Webex. Mit Hilfe der Aufnahme werden wir das Interview transkribieren und hinterher auswerten. Bei der Transkription werden alle personenbezogenen Daten, die eventuell im Interview geäußert werden, anonymisiert, sodass keine Rückschlüsse auf Sie oder andere Personen möglich sind. Alle anderen personenbezogenen Daten (u.a. Ihre Kontaktdaten) und auch die Audioaufnahmen werden nach der Transkription und Qualitätssicherung der Transkripte gelöscht, nicht an unbefugte Dritte weitergegeben, vervielfältigt oder in dieser Form veröffentlicht.

Ihre Teilnahme an dem Interview ist absolut freiwillig und Sie können Ihre Einwilligung zur Teilnahme jederzeit widerrufen. Haben Sie vor Beginn der Aufnahme noch Fragen?

**Wenn keine Rückfragen:* Dann würde ich die Aufnahme jetzt starten* *(Aufnahme starten)*

Intro

Zu Beginn würde uns erstmal interessieren, welche Rolle Sie in Ihrer Einrichtung haben, wie Sie in die Umsetzung der Impfpflicht eingebunden sind und ob auch noch weitere Personen bei Ihnen in der Einrichtung da mitarbeiten.

---

1. **Prozess** → Wie wird die einrichtungsbezogene COVID-19 Impfpflicht von Einrichtungsleitungen und durch Gesundheitsämter umgesetzt?

Zunächst würde uns interessieren, wie das bei Ihnen mit der Impfpflicht abgelaufen ist.

- Wie (und wann?) haben Sie von der Impfpflicht erfahren? Können Sie sich noch daran erinnern?
  - Haben Sie sich irgendwie auf die Impfpflicht vorbereitet? Wie?
  - Gab es Unterstützung vom Bundesgesundheitsministerium oder von den Gesundheitsministerien des Landes und wie sah diese aus?
  - Haben Sie sich zur Umsetzung der Impfpflicht mit anderen Pflegeeinrichtungen oder auf Landesebene ausgetauscht?
  - Hatten Sie Fragen zur Impfpflicht? Wem konnten Sie diese stellen?

- Sie hatten ja schon angedeutet, dass unterschiedliche Personen in die Umsetzung der Impfpflicht eingebunden sind: Welche Stellen sind denn bei Ihnen konkret mit der Umsetzung der Impfpflicht beschäftigt?
  - **Und welche Stelle übernimmt da welche Aufgaben? (zum Beispiel die Kontrolle der Nachweise, die Information über die Impfpflicht an die Mitarbeitenden, …)**
  - Ist die Rollenzuweisung eindeutig? Weiß jeder was er*sie zu tun hat?
    Hier darauf hinweisen, dass es nicht um Namen geht

- Nun wollen wir ein bisschen mehr darüber erfahren, wie Sie die Impfpflicht konkret bei sich vor Ort umgesetzt haben. Erzählen Sie doch mal.
  - Wie haben Sie die Impfpflicht im Haus an die Mitarbeitenden kommuniziert? (Gab es diesbezüglich spezifische Infoveranstaltungen, Anleitungen?)
  - **Woher wissen Sie, ob die Mitarbeitenden geimpft sind oder nicht?**
    - **Und ab wann wurden die Nachweise kontrolliert?**
  - **Wie und wann wurden Gesundheitsämter mit eingebunden?**
  - **Was wurde denn gemacht, wenn jemand keinen Impfnachweis vorlegen konnte?**
    - **Kam es zu Kündigungen, wurden Mitarbeitende versetzt oder welche anderen Maßnahmen wurden getroffen?**
    - **Wie haben Sie zur Impfung motiviert?**

- **Wie läuft es ab, nun da seit 01. Oktober nicht mehr zwei, sondern drei Impfungen notwendig sind: finden erneut Kontrollen bei Ihnen statt oder wie gehen Sie damit um?**

---

2. Herausforderungen → Welche Herausforderungen gibt es in der Umsetzung der einrichtungsbezogenen COVID-19 Impfpflicht?

Vielen Dank, das waren ja schon mal sehr spannende Einblicke! Als nächstes interessiert uns, ob Sie Herausforderungen im Zusammenhang mit der Impfpflicht erlebt haben…

- Dazu erstmal ganz offen gefragt: Welche Herausforderungen rund um die Impflicht kommen Ihnen da als erstes in den Sinn? Welche haben Sie selbst erlebt?
  - **Was würden Sie sagen, welche war die größte Herausforderung?**
  - Wie haben Sie die Unterstützung durch den Bund, das Land und das Gesundheitsamt empfunden?
  - Gab es bei Ihnen im Haus genügend personelle Ressourcen für die Umsetzung? Hätten Sie noch etwas anderes gebraucht?
  - Und wie würden Sie die Kommunikation zur Impfpflicht an die Mitarbeitenden bewerten? War sie gelungen oder wie würden Sie diese verbessern?
  - **Welche Erfahrungen haben Sie mit Mitarbeitenden gemacht, die keinen Nachweis erbringen können?**
    - **Wie war Ihre Erfahrung mit Gefälligkeitsattesten oder Verzögerungstaktiken? Können Sie davon berichten?**

- **Was würden Sie sagen, was waren oder sind aus Ihrer Sicht gute Strategien im Umgang mit … (gewesen)?** **Hier konkret nach den Strategien im Umgang mit den Herausforderungen fragen, die im vorherigen Gespräch besonders prominent/ relevant waren**
  - Fehlender Unterstützung durch Bund und Land
  - Gefälligkeitsattesten und Verzögerungstaktiken
  - Impfkritischen oder verärgerten Mitarbeitenden (insbesondere nach der Übergangsfrist)
  - Fehlenden Ressourcen
  - Situativ Strategien im Umgang mit weiteren Herausforderungen…

---

3. Weitere Bereiche → Welche Effekte hat die einrichtungsbezogene COVID-19 Impfpflicht auf andere Bereiche?

Wir haben uns jetzt ausführlich darüber unterhalten, wie die Impfpflicht bei Ihnen in der Einrichtung abläuft und umgesetzt wird, welche Herausforderungen Sie erlebt haben und wie Sie diese bewältigt haben. So eine Impfpflicht kann sich ja auch auf andere Bereiche des Arbeitslebens auswirken.

- Hat sich aus Ihrer Sicht seit der Einführung der Impfpflicht, also seit März dieses Jahres, etwas in Ihrer Einrichtung verändert? Wir denken da an das Miteinander. Können Sie das beschreiben?
    - Wie gehen geimpfte und ungeimpfte Mitarbeitende miteinander um?
    - **Was haben Sie beobachtet, wie haben die bereits geimpften Mitarbeitenden auf die Einführung der Impfpflicht reagiert?**
    - **Wie haben ungeimpfte Mitarbeitende auf die Einführung der Impfpflicht reagiert?**

- Gab es Dinge, die in Ihrer Einrichtung auf Grund der Impfpflicht liegen geblieben sind? Welche sind das?
    - Betrifft das auch Ihre Arbeit?
    - Welche Auswirkungen hatte das oder könnte das haben?
    - Haben sich Ihre eigentlichen Arbeitsaufgaben mit den neuen Aufgaben zur Impfpflicht verändert?

- War die Impfpflicht auch bei Ihren Patient:innen ein Thema? Inwiefern?
    - Haben Patient:innen sich erkundigt, ob die Mitarbeitenden geimpft sind?
    - **Und für Sie selbst gesprochen, ist der Schutz der Patient:innen ein guter Grund für eine Impfpflicht oder eher unnötig, warum**?

Outro

Die Impfpflicht wird ja zum 01.01.2023 voraussichtlich auslaufen.

- Was halten Sie davon?
- Unterstützen Sie das oder nicht?
- Warum?

Vielen Dank! Wir sind nun fast am Ende unseres Interviews angekommen. Deshalb möchte ich Ihnen jetzt die Gelegenheit geben:

- Liegt Ihnen noch etwas auf dem Herzen, was Sie gerne loswerden möchten? Haben wir etwas Wichtiges nicht angesprochen?

Verabschiedung

Vielen Dank für das interessante Gespräch, für die Einblicke in Ihre Arbeit und Ihre persönlichen Sichtweisen zur Impfpflicht.

- Gerne lassen wir Ihnen die Ergebnisse der Interviews als Kurzbericht zukommen… Wenn Sie daran interessiert sind, schicken Sie uns bitte einfach eine kurze Mail an xxx@rki.de
- Für die Überweisung der Aufwandsentschädigung…

Wir wünschen Ihnen noch einen schönen Tag!
